# Supplementary material for: Novel NADPH Oxidase-2 Inhibitors as Potential Anti-Inflammatory and Neuroprotective Agents
Source: Antioxidants (Basel). 2023 Aug 23;12(9):1660. doi: 10.3390/antiox12091660 (PMC10525516; doi:10.3390/antiox12091660)
Supplement: Supplementary file 1 [file antioxidants-12-01660-s001.zip › antioxidants-2543440-supplementary.pdf]

Supporting Information

Novel NADPH oxidase-2 inhibitors as potential anti-inflammatory and neuroprotective agents

Matea Juric [1,†], Varun Rawat [2,†], Radhika Amaradhi [2,#], Jacek Zielonka [1,*] and Thota Ganesh [2,*]

1 Department of Biophysics, Medical College of Wisconsin, Milwaukee, WI, USA; jzielonk@mcw.edu, mjuric@mcw.edu
2 Department of Pharmacology and Chemical Biology, Emory University School of Medicine, Atlanta, GA, USA; tganesh@emory.edu, varun.rawat@emory.edu, radhika.amaradhi@utsa.edu
* Correspondence: T.G. tganesh@emory.edu and J.Z. jzielonk@mcw.edu
† These authors contributed equally to this work.
# Current address: Center for Innovative Drug Discovery, University of Texas at San Antonio, San Antonio, TX.

Table of Contents:

1. General analytical procedure……………………………………………………………2
2. Synthesis of compounds TG15-131•HCl and TG15-124•HCl………………………………2
3. Synthesis of compounds TG15-132•HCl and TG15-139•HCl………………………………3
4. Synthesis of compound TG17-55•2HCl…………………………………………………4
5. Synthesis of compounds TG17-57 and TG17-56…………………………………………5
6. Synthesis of compound TG15-293……………………………………………………6
7. ^{1}H NMR, ^{19}F NMR, MS spectra and HPLC chromatograms of compounds……………8-29

1. General experimental procedure

Proton NMR spectra were recorded in DMSO-d_6/$CDCl_3$ on Varian and Inova-400 spectrometers (400 MHz). Thin layer chromatography was performed on pre-coated, aluminum-backed plates (silica gel 60 F_{254}, 0.25 mm thickness) from EM Science and was visualized by UV lamp, phosphomolybdic acid solution and ninhydrin. Column chromatography was performed with silica gel cartridges on Teledyne-ISCO CombiFlash instrument. Agilent LC-MS system was used to determine the mass and purity of the products. LC-MS conditions: mobile phase A: methanol (0.1% acetic acid); mobile phase B: water (0.1% acetic acid); column: ZORBAX Eclipse XDB C18 5 µm, 4.6 mm × 150 mm. Gradient B 80% at 0 min, linearly decreased to 5% by 7 min, and then linear increase to 40% by 12 min; UV wavelength = 254 nm; flow rate = 1 mL/min. Furthermore, purity of several key compounds was determined by Water's HPLC instrument. HPLC Conditions: mobile phase A: water (0.1% trifluoroacetic acid); mobile phase B: acetonitrile (0.1% trifluoroacetic acid); column: XBridge C18 5 µm, 4.6 mm × 150 mm; gradient: 10% B at 0 min, increased linearly to 90% by 10 min, then decreased to 10% by 12 min; UV wavelength = 230 nm; flow rate = 1 mL/min. Compounds with >95% purity by HPLC were tested in cellular bioassays and for DMPK properties. Compounds **1** and **2** were reported in the literature and the characterization data for these derivatives was in good agreement with the literature data (see ref. 23)

2. Synthesis of compounds TG15-131•HCl and TG15-124•HCl

To a solution of **1** (0.1 g, 0.31 mmol, 1 equiv.) in a mixture of tetrahydrofuran (THF) and methanol (1:1, 10 mL) was added K_2CO_3 (0.17 g, 1.24 mmol, 4 equiv.) followed by bromo derivative **3** (0.1 g, 0.41 mmol, 1.3 equiv.) and allowed to stir at room temperature for 48 h. Solvent was evaporated. To a crude compound was added water (5 mL) and extracted with ethyl acetate (2 × 10 mL). A combined organic layer was concentrated and purified on a CombiFlash system using 5-6% methanol and dichloromethane and after solvent evaporation the compound **TG15-131** was obtained as a solid. To a solution of **TG15-131** (0.05 g, 0.12 mmol, 1 equiv.) in methanol (3 mL)

was added aq. HCl (1 M, 0.35 mL, 0.35 mmol, 2.5 equiv.) and allowed to stir at room temperature for 3 h. The solid precipitated and was filtered, triturated with ethyl acetate (5 mL) and dried to get the required compound **TG15-131•HCl** as a solid. (Yield: 62% over two steps). Individual step yields are shown in the Scheme 1.

Compound **TG15-124•HCl** was synthesized following this method beginning with a chlorinated starting material **2** (Scheme 1, see also ref. 23).

*3-(3-(Dimethylamino)propyl)-6-fluoro-2-((2-(pyridine-2-yl)ethyl)thio)quinazolin-4(3H)-one hydrochloride (**TG15-131•HCl**)*: ^{1}H NMR (400 MHz, DMSO-d_6): δ 10.88 (s, 1H), 8.77 (d, *J* = 5.6 Hz, 1H), 8.45 (t, *J* = 7.7 Hz, 1H), 8.06 (d, *J* = 7.9 Hz, 1H), 7.81 – 7.64 (m, 4H), 4.07 (t, *J* = 6.8 Hz, 2H), 3.80 (t, *J* = 6.5 Hz, 2H), 3.59 – 3.52 (m, 2H), 3.12 (dd, *J* = 8 Hz, 4.8 Hz, 2H), 2.71 (d, *J* = 4.7 Hz, 6H), 2.17 – 2.04 (m, 2H); ^{19}F NMR (376 MHz, DMSO-d_6): δ -114.23 (dt, *J* = 13.5, 6.8 Hz); LCMS (ESI): LCMS (ESI): >96% purity; *m/z*, 387 [(M-HCl) + H]$^{+}$; HPLC purity: 98.9%.

*3-(6-Chloro-4-oxo-2-((2-(pyridine-2-yl)ethyl)thio)quinazolin-3(4H)-yl)-1-(dimethyl-l4-azaneyl) propan-1-ylium hydrochloride (**TG15-124•HCl**):* ^{1}H NMR (400 MHz, DMSO-d_6): δ 10.68 (s, 1H), 8.76 (d, *J* = 5.4 Hz, 1H), 8.38 (t, *J* = 7.4 Hz, 1H), 8.02- 7.88 (m, 2H), 7.84 (dd, *J* = 8.7, 2.5 Hz, 1H), 7.75 (t, *J* = 6.4 Hz, 1H), 7.62 (d, *J* = 8.7 Hz, 1H), 4.07 (t, *J* = 6.9 Hz, 2H), 3.79 (t, *J* = 6.6 Hz, 2H), 3.53 (t, *J* = 6.5 Hz, 2H), 3.12 (dt, *J* = 10.3, 5.2 Hz, 2H), 2.71 (d, *J* = 4.7 Hz, 6H), 2.15 – 2.04 (m, 2H); LCMS (ESI): >95% purity; *m/z*, 403 [(M-HCl) + H]$^{+}$; HPLC purity: 99.3%.

3. Synthesis of compounds TG15-132•HCl and TG15-139•HCl

To a solution of **1** (0.25 g, 0.8 mmol, 0.9 equiv.) in a mixture of THF and methanol (1:1, 15 mL) was added K_2CO_3 (0.49 g, 3.57 mmol, 4 equiv.) followed by bromo derivative **4** (0.2 g, 0.89 mmol, 1 equiv.) and allowed to stir at room temperature for 48 h. Solvent was evaporated. To a crude compound was added water (10 mL) and extracted with ethyl acetate (2 × 10 mL). Combined organic layer was concentrated and purified on a CombiFlash system using 4-5% methanol and

dichloromethane to obtain the compound **TG15-132** as a solid. To a solution of **TG15-132** (0.58 g, 1.36 mmol, 1 equiv.) in methanol (6 mL) aq. HCl (1M, 3.4 mL, 3.4 mmol, 2.5 equiv.) was added and stirred at room temperature for 3 h. The solid precipitated and was filtered, triturated with ethyl acetate (5 mL) and dried to obtain **TG15-132•HCl** as a solid. Yield: 61% over two steps). Individual step yields are shown in Scheme 1.

Compound **TG15-139•HCl** was synthesized following the same protocol, but beginning with a chlorinated starting material **2**.

*2-((2-(1H-Indol-3-yl)ethyl)thio)-3-(3-(dimethylamino)propyl)-6-fluoroquinazolin-4(3H)-one hydrochloride (**TG15-132•HCl**)*: ^{1}H NMR (400 MHz, DMSO-d_6): δ 10.95 (s, 1H), 10.11 (s, 1H), 7.80 – 7.64 (m, 4H), 7.36 (d, *J* = 8.0 Hz, 1H), 7.28 (d, *J* = 2.2 Hz, 1H), 7.13 – 7.00 (m, 2H), 4.13 (t, *J* = 7.0 Hz, 2H), 3.58 (t, *J* = 7.5 Hz, 2H), 3.20 – 3.09 (m, 4H), 2.72 (s, 6H), 2.14 – 2.04 (m, 2H); ^{19}F NMR (376 MHz, DMSO-d_6): δ -114.46 (td, *J* = 8.4, 5.1 Hz); LCMS (ESI): LCMS (ESI): >95% purity; *m/z*, 425 [(M-HCl) + H]$^+$; HPLC purity: 96.4%.

*2-((2-(1H-Indol-3-yl)ethyl)thio)-6-chloro-3-(3-(dimethylamino)propyl)quinazolin-4(3H)-one hydrochloride (**TG15-139•HCl**):* ^{1}H NMR (400 MHz, DMSO-d_6): δ 10.95 (s, 1H), 10.24 (s, 1H), 8.03 (d, *J* = 2.5 Hz, 1H), 7.86 (dd, *J* = 8.7, 2.5 Hz, 1H), 7.73 – 7.58 (m, 2H), 7.36 (d, *J* = 7.9 Hz, 1H), 7.28 (d, *J* = 2.0 Hz, 1H), 7.13 – 6.98 (m, 2H), 4.12 (t, *J* = 6.9 Hz, 2H), 3.67 – 3.48 (m, 2H), 3.25 – 3.06 (m, 4H), 2.71 (s, 6H), 2.20 – 1.99 (m, 2H); LCMS (ESI): LCMS (ESI): >95% purity; *m/z*, 440 [(M-HCl) + H]$^+$; HPLC purity: 95.7%.

4. Synthesis of compound TG17-55•2HCl

To a solution of **1** (0.15 g, 0.53 mmol, 1 equiv.) and 3-(N,N-dimethyl)-1-bromo propane (see Scheme 1) (0.19 g, 0.8 mmol, 1.5 equiv.) in a mixture of THF and methanol (10 mL, 1:1) was added K_2CO_3 (0.22 g, 1.6 mmol, 3 equiv.) and stirred at room temperature for 24 h. Solvent was evaporated, water (10 mL) was added to the reaction mixture and the product was extracted with

10% methanol and dichloromethane (2 × 10 mL). The combined organic layer was dried over Na_2SO_4 and concentrated and taken to next step. To a solution of above product (0.15 g, 0.41 mmol, 1 equiv.) in dichloromethane (5 mL), 4 M HCl (0.3 mL, 1.23 mmol, 3 equiv.) was added and allowed to stir at room temperature for 12 h. The precipitated solid was washed with dichloromethane (10 mL) followed by ethyl acetate (10 mL) and dried in a vacuum to get the product **TG17-55•2HCl** (Yield: 51% over two steps)

*3-(3-(Dimethylamino)propyl)-2-((3-(dimethylamino)propyl)thio)-6-fluoroquinazolin-4(3H)-one dihydrochloride (**TG17-55.2HCl**)***:** ^{1}H NMR (400 MHz, DMSO-d_6): δ 10.69 (s, 1H), 10.55 (s, 1H), 7.79 – 7.75 (m, 1H), 7.74 – 7.68 (m, 2H), 4.14 (t, *J* = 7.0 Hz, 2H), 3.39 (d, *J* = 7.0 Hz, 2H), 3.28 – 3.04 (m, 4H), 2.75 (dd, *J* = 9.2, 4.5 Hz, 12H), 2.25 – 2.08 (m, 4H); ^{19}F NMR (376 MHz, DMSO-d_6): δ -114.30 (td, *J* = 8.2, 5.4 Hz); *m/z*, 367 [(M-2HCl) + H]$^+$; Purity from HPLC: 99.5%.

5. Synthesis of compounds TG17-57 and TG17-56

To a solution of 5-fluoroisatoic anhydride **5** (0.97 g, 5.4 mmol, 1 equiv.) in dioxane (20 mL) was added amine **6** (1 g, 5.4 mmol, 1 equiv.) and stirred at room temperature for 2 h. To the reaction mixture was added water (10 mL) and extracted with ethyl acetate (2 × 15 mL). Combined organic layer was dried over sodium sulfate, concentrated, and taken for next step. To a solution the obtained amine (2 g, 6.21 mmol, 1 equiv.) in ethanol (20 mL) were added NaOH (0.37 g, 9.3 mmol, 1.5 equiv.) followed by CS_2 (0.56 mL, 9.3 mmol, 1.5 equiv.) and refluxed for 20 h. Reaction mixture was cooled to room temperature, added water (10 mL) and extracted with 10% methanol/dichloromethane (2 × 10 mL). The combined organic layer was dried over Na_2SO_4, concentrated, and purified on CombiFlash system using 5-6% methanol and dichloromethane to get the required cyclized product **8a**. (Yield: 46% over two steps). To a solution of **8a** (0.4 g, 1.09 mmol, 1 equiv.) and **4** (see Scheme 1) (0.31 g, 1.42 mmol, 1.3 equiv.) in THF and methanol (15 mL, 1:1) was added K_2CO_3 (0.45 g, 3.29 mmol, 3 equiv.) and stirred at room temperature for 48 h. Solvent was evaporated, water (10 mL) was added to the reaction mixture and extracted with

ethyl acetate (2 × 10 mL). The combined organic layer was dried over Na_2SO_4 and concentrated and purified on CombiFlash using 5-6% methanol and dichloromethane to get product **TG17-57**. (Yield: 52%)

Compound **TG17-56** was synthesized following the same protocol, but beginning with amine starting material **7** (Scheme 2). Characterization data is shown below.

*2-((2-(1H-Indol-3-yl)ethyl)thio)-6-fluoro-3-(3-(4-isopropylpiperazin-1-yl)propyl)quinazolin-4(3H)-one (**TG17-57**):* ^{1}H NMR (400 MHz, DMSO-d_6): δ 10.90 (d, *J* = 2.4 Hz, 1H), 7.76 (dd, *J* = 8.7, 2.9 Hz, 1H), 7.72 – 7.62 (m, 3H), 7.40 – 7.33 (m, 1H), 7.26 (d, *J* = 2.3 Hz, 1H), 7.14 – 6.97 (m, 2H), 4.19 – 4.04 (m, 2H), 3.57 (dd, *J* = 8.4, 6.7 Hz, 2H), 3.16 (t, *J* = 7.5 Hz, 2H), 2.52 (s, 1H), 2.26 (s, 10H), 1.83 (p, *J* = 6.4 Hz, 2H), 0.88 (d, *J* = 6.4 Hz, 6H); ^{19}F NMR (376 MHz, DMSO-d_6): δ -114.82 (q, *J* = 7.6 Hz); *m/z*, 508 [M + H]$^+$; Purity by HPLC: 98%

*2-((2-(1H-Indol-3-yl)ethyl)thio)-3-(3-(4-ethylpiperazin-1-yl)propyl)-6-fluoroquinazolin-4(3H)-one (**TG17-56**):* ^{1}H NMR (400 MHz, DMSO-d_6): δ 10.91 (d, *J* = 2.5 Hz, 1H), 7.79 – 7.61 (m, 4H), 7.36 (d, *J* = 8.0 Hz, 1H), 7.25 (d, *J* = 2.3 Hz, 1H), 7.14 – 6.99 (m, 2H), 4.14 – 4.06 (m, 2H), 3.57 (dd, *J* = 8.4, 6.6 Hz, 2H), 3.16 (t, *J* = 7.5 Hz, 2H), 2.47 – 2.09 (m, 12H), 1.83 (q, *J* = 7.0 Hz, 2H), 1.01 – 0.92 (m, 3H); ^{19}F NMR (376 MHz, DMSO-d_6): δ -114.73; *m/z*, 494 [M + H]$^+$; Purity by HPLC: 98.9%

6. Synthesis of compound TG15-293

To the salt solution of **9** (Scheme 2) (3.31 g, 21.6 mmol, 1 equiv.) in dioxane (30 mL), triethyl amine (3.8 mL, 28.1 mmol, 1.3 equiv.) was added and stirred at room temperature for 10 minutes. Then, **5** was added to the reaction mixture and stirred at room temperature for 2 h. To this reaction mixture was added water (15 mL) and extracted with ethyl acetate (2 × 15 mL). Combined organic layer was dried over sodium sulfate, concentrated to get a crude amine and proceeded for next step. To a solution of amine (4.3 g, 16.9 mmol, 1 equiv.) in ethanol (35 mL) were added NaOH (1 g, 25.3 mmol, 1.5 equiv.) followed by CS_2 (1.53 mL, 25.3 mmol, 1.5 equiv.) and refluxed for 24 h.

Reaction mixture was cooled to room temperature, precipitated solid was filtered, washed with methanol (10 mL) and dried under vacuum to get the required cyclized product **10**, which was taken to the next step without purification (Yield: 69% over two steps).

To a solution of **10** (0.5 g, 1.68 mmol, 1 equiv.) and bromo derivative **4** (0.41 g, 1.85 mmol, 1.1 equiv.) in THF and methanol (10 mL, 1:1) K_2CO_3 (0.7 g, 5.06 mmol, 3 equiv.) was added and stirred at room temperature for 48 h. Solvent was evaporated, water (10 mL) was added to the reaction mixture and extracted with ethyl acetate (2 x 10 mL). The combined organic layer was dried over Na_2SO_4 and concentrated and purified on CombiFlash system using 5-6% methanol and dichloromethane to obtain the product **11** (Yield: 48%)

To a solution of **11** (0.36 g, 0.81 mmol, 1 equiv.) in THF and water (10 mL, 7:3), LiOH (0.08 g, 2.04 mmol, 2.5 equiv.) was added and stirred at room temperature for 2 h. Reaction mixture was acidified to pH 4 using 1 N HCl and extracted with ethyl acetate (2 x 10 mL). Combined organic later was dried over Na_2SO_4 and concentrated to afford the crude acid precursor, which was taken to next step without purification. To a solution of acid (0.12 g, 0.2 mmol, 1 equiv.) and commercially available, 1-isopropylpiperazine **12** (0.04 g, 0.2 mmol, 1 equiv.) in DMF (10 mL) DMAP (catalytic) and EDCI (0.07 g, 0.26 mmol, 1.3 equiv.) was added and stirred at room temperature for 24 h. To the reaction mixture was added ethyl acetate (15 mL) and washed with saturated aqueous ammonium chloride (10 mL), saturated sodium bicarbonate solution (10 mL) followed by aqueous sodium chloride solution (10 mL). The organic layer was dried over Na_2SO_4, concentrated, and purified on CombiFlash system using 3-4% methanol and dichloromethane to get the required coupled product **TG15-293** (Yield: 66%, over two steps).

*2-((2-(1H-Indol-3-yl)ethyl)thio)-6-fluoro-3-(4-(4-isopropylpiperazin-1-yl)-4-oxobutyl)quinazolin-4(3H)-one (**TG15-293**).* ^{1}H NMR (400 MHz, DMSO-d_6): δ 10.89 (s, 1H), 7.79 – 7.58 (m, 4H), 7.36 (d, *J* = 7.1 Hz, 1H), 7.26 (d, *J* = 2.3 Hz, 1H), 7.13 – 6.97 (m, 2H), 4.16 – 4.01 (m, 2H), 3.59 – 3.48 (m, 2H), 3.36 (dt, *J* = 9.2, 5.5 Hz, 4H), 3.21 – 3.11 (m, 2H), 2.64 (p, *J* = 6.5 Hz, 1H), 2.40 (t, *J* =

6.9 Hz, 4H), 2.35 – 2.28 (m, 2H), 1.92 (p, *J* = 7.1 Hz, 2H), 0.95 (d, *J* = 6.5 Hz, 6H); ^{19}F NMR (376 MHz, DMSO-d_6): δ -114.74 (td, *J* = 8.5, 5.2 Hz); *m/z*, 536 $[M + H]^+$; Purity from HPLC: 98.2%.

7. ^{1}H NMR, ^{19}F NMR, MS spectra and HPLC chromatograms of compounds

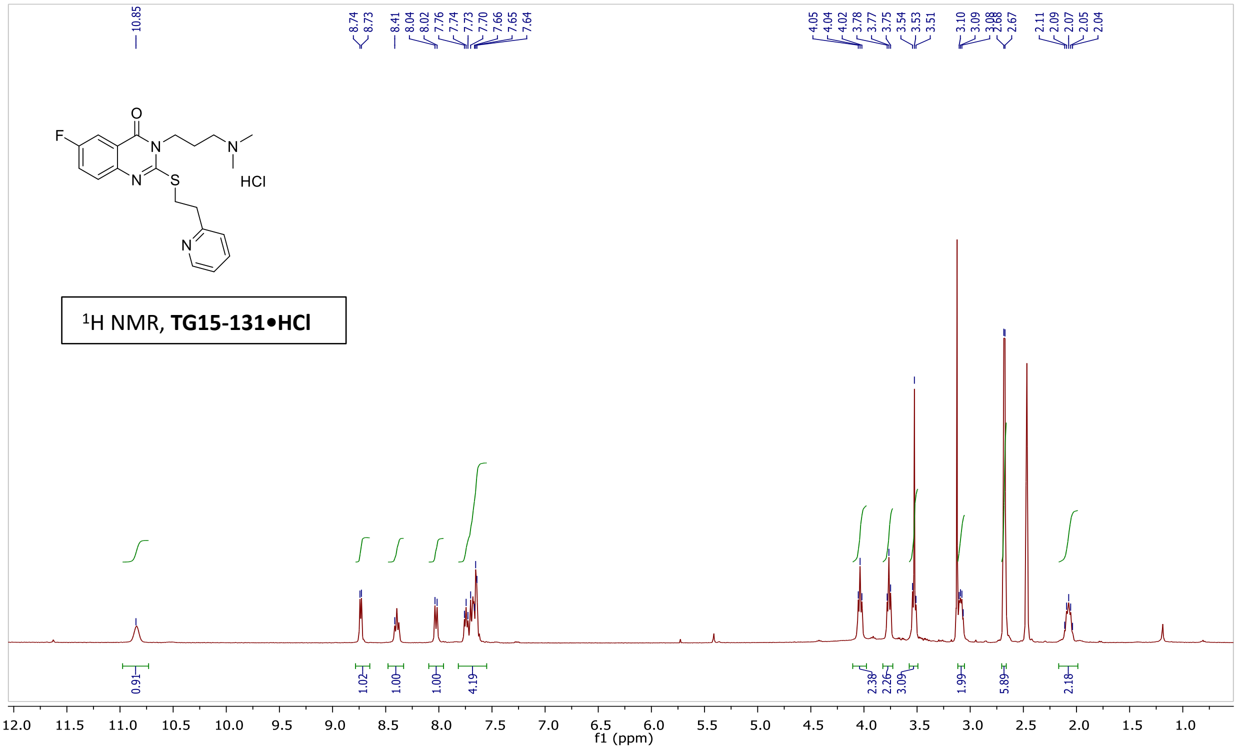

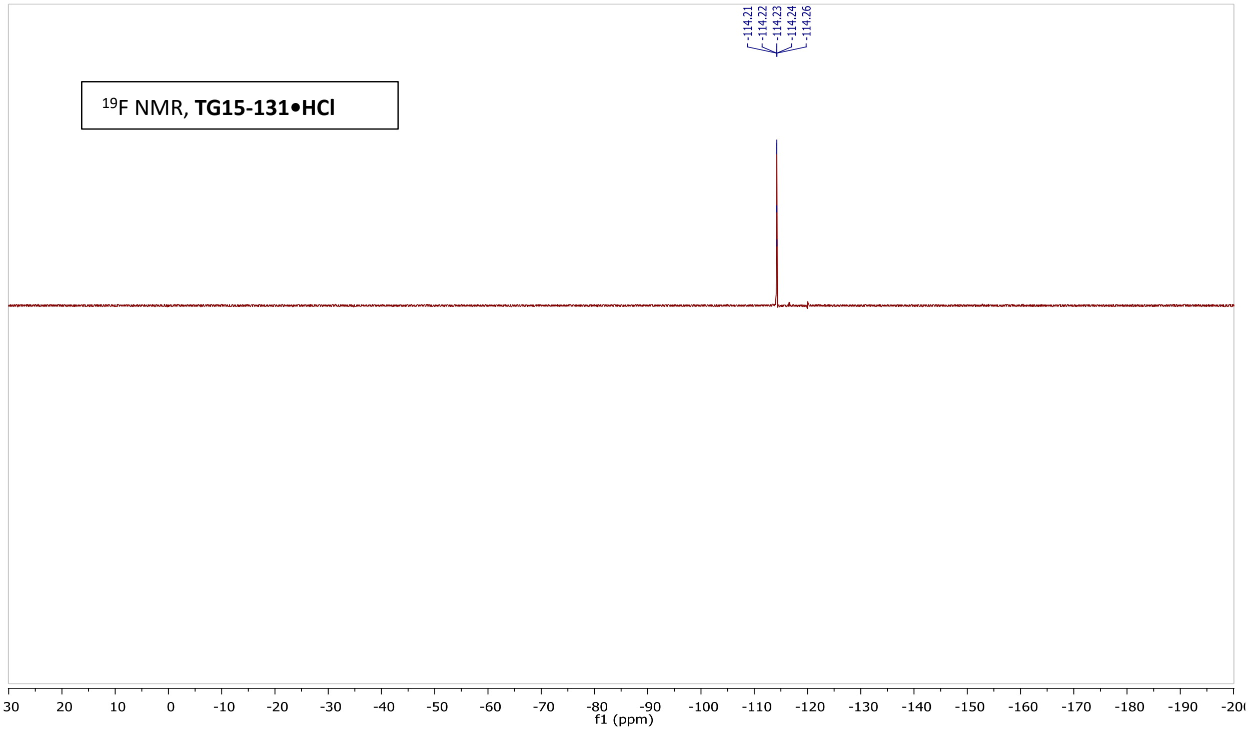
19F NMR, TG15-131•HCl
-114.21
-114.22
-114.23
-114.24
-114.26
f1 (ppm)

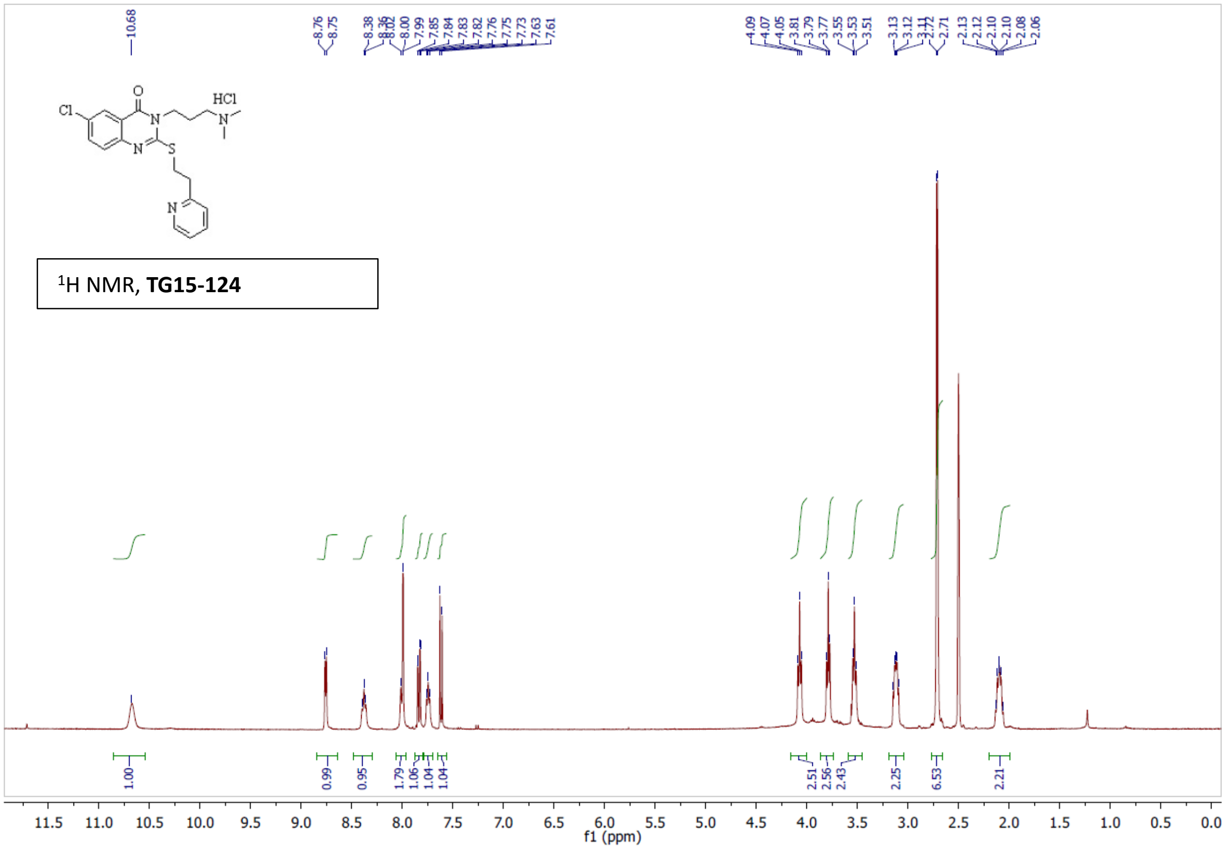
1H NMR, TG15-124
HCl
Cl
O
N
S
10.68
8.76
8.75
8.38
7.99
7.85
7.84
7.83
7.82
7.76
7.75
7.73
7.63
7.61
4.09
4.07
4.05
3.81
3.79
3.77
3.55
3.53
3.51
3.13
3.12
3.11
2.71
2.13
2.12
2.10
2.10
2.08
2.06
1.00
0.99
0.95
1.79
1.06
1.04
1.04
2.51
2.56
2.43
2.25
6.53
2.21
f1 (ppm)

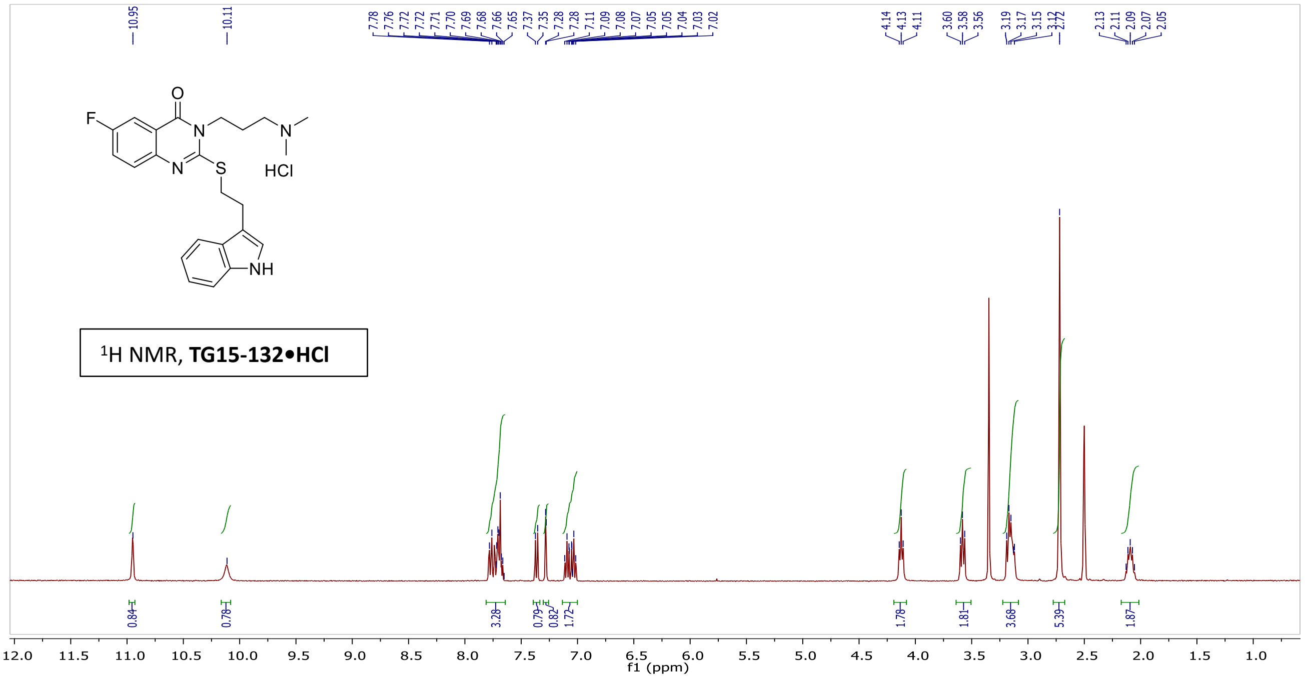
F
O
N
N
S
N
HCl
NH
^{1}H NMR, **TG15-132•HCl**
10.95
10.11
7.78 7.76 7.72 7.72 7.71 7.70 7.69 7.68 7.66 7.65 7.37 7.35 7.28 7.28 7.11 7.09 7.08 7.07 7.05 7.05 7.04 7.03 7.02
4.14 4.13 4.11
3.60 3.58 3.56
3.19 3.17 3.15 3.12 2.72
2.13 2.11 2.09 2.07 2.05
0.84 0.78 3.28 0.79 0.82 1.72 1.78 1.81 3.68 5.39 1.87
12.0 11.5 11.0 10.5 10.0 9.5 9.0 8.5 8.0 7.5 7.0 6.5 6.0 5.5 5.0 4.5 4.0 3.5 3.0 2.5 2.0 1.5 1.0
f1 (ppm)

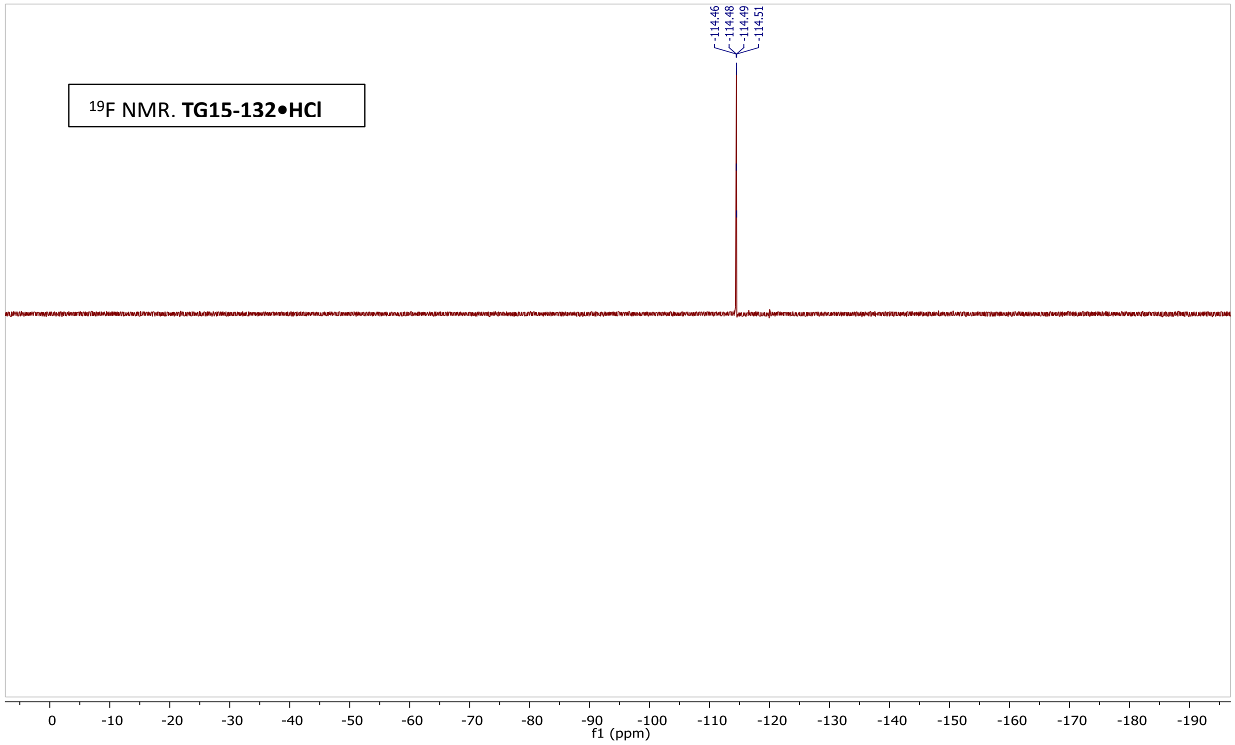
^{19}F NMR. **TG15-132•HCl**
-114.46 -114.48 -114.49 -114.51
0 -10 -20 -30 -40 -50 -60 -70 -80 -90 -100 -110 -120 -130 -140 -150 -160 -170 -180 -190
f1 (ppm)

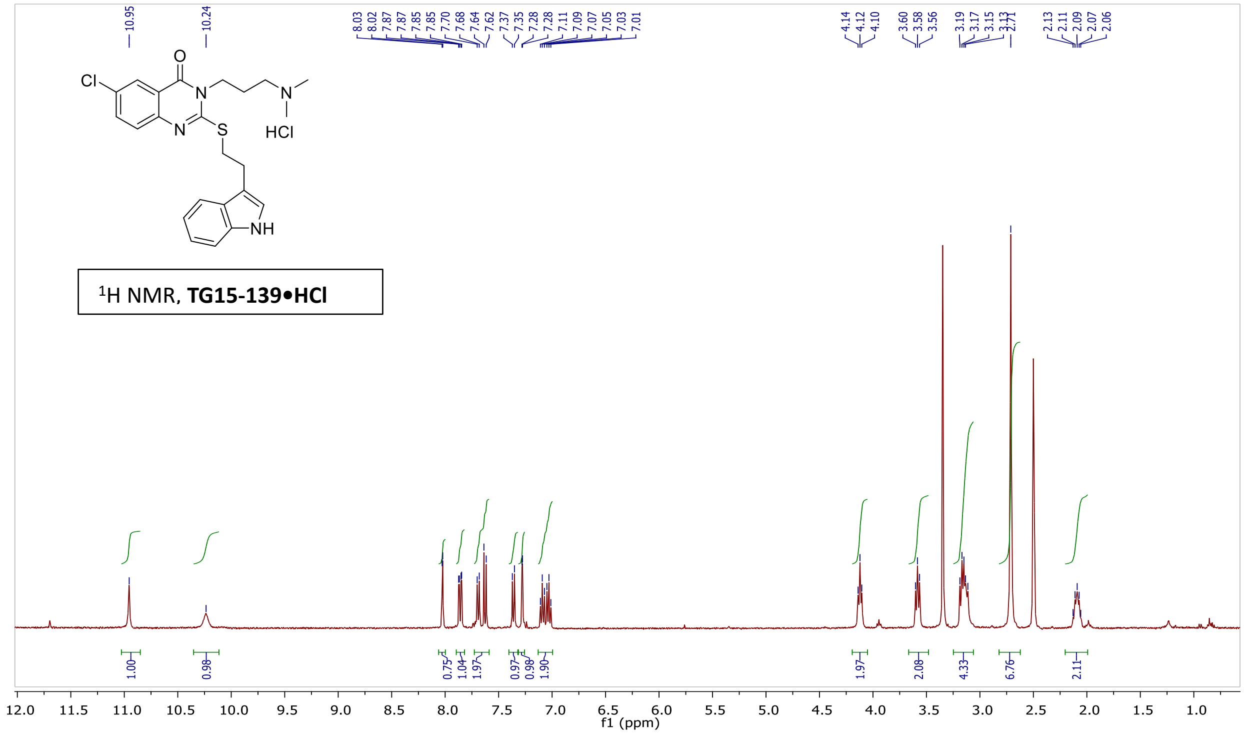

Cl
O
N
N
S
N
HCl
NH
1H NMR, TG15-139•HCl
f1 (ppm)

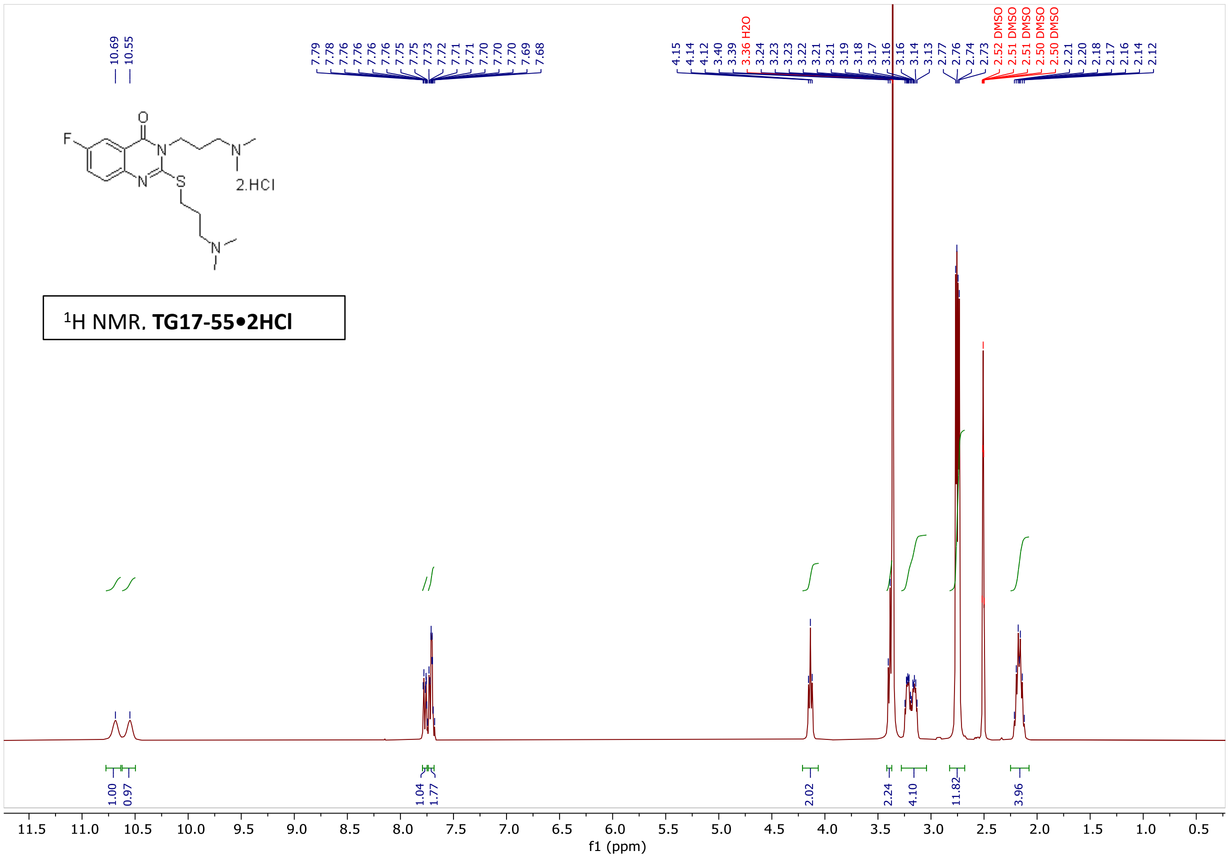

^{1}H NMR. **TG17-55•2HCl**

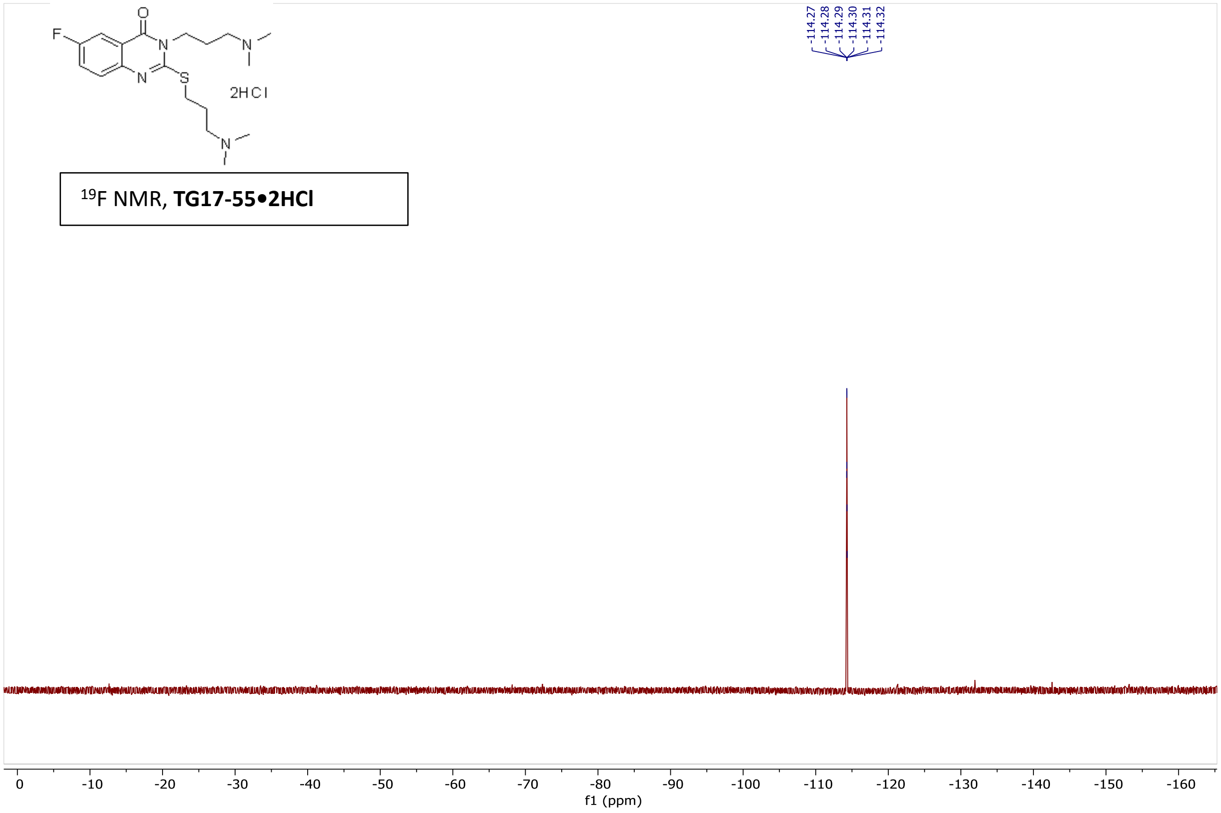

^{19}F NMR, **TG17-55•2HCl**

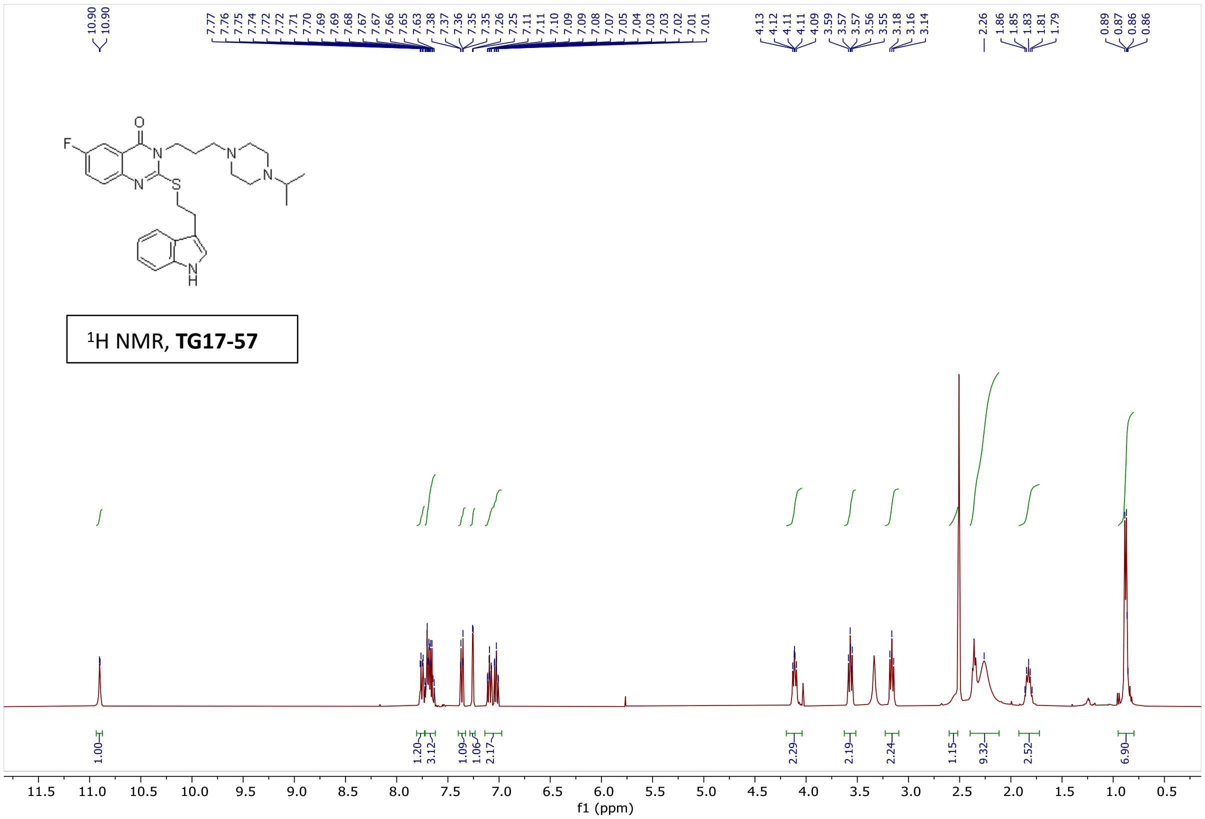
10.90
10.90
7.77 7.76 7.75 7.74 7.72 7.72 7.71 7.70 7.69 7.69 7.68 7.67 7.67 7.66 7.65 7.63 7.38 7.37 7.36 7.35 7.35 7.26 7.25 7.11 7.11 7.10 7.09 7.09 7.08 7.07 7.05 7.04 7.03 7.03 7.02 7.01 7.01
4.13 4.12 4.11 4.11 4.09 3.59 3.57 3.57 3.56 3.55 3.18 3.16 3.14
2.26 1.86 1.85 1.83 1.81 1.79
0.89 0.87 0.86 0.86
F
O
N
N
S
N
N
N
H
1.00
1.20
3.12
1.09
1.06
2.17
2.29
2.19
2.24
1.15
9.32
2.52
6.90
f1 (ppm)

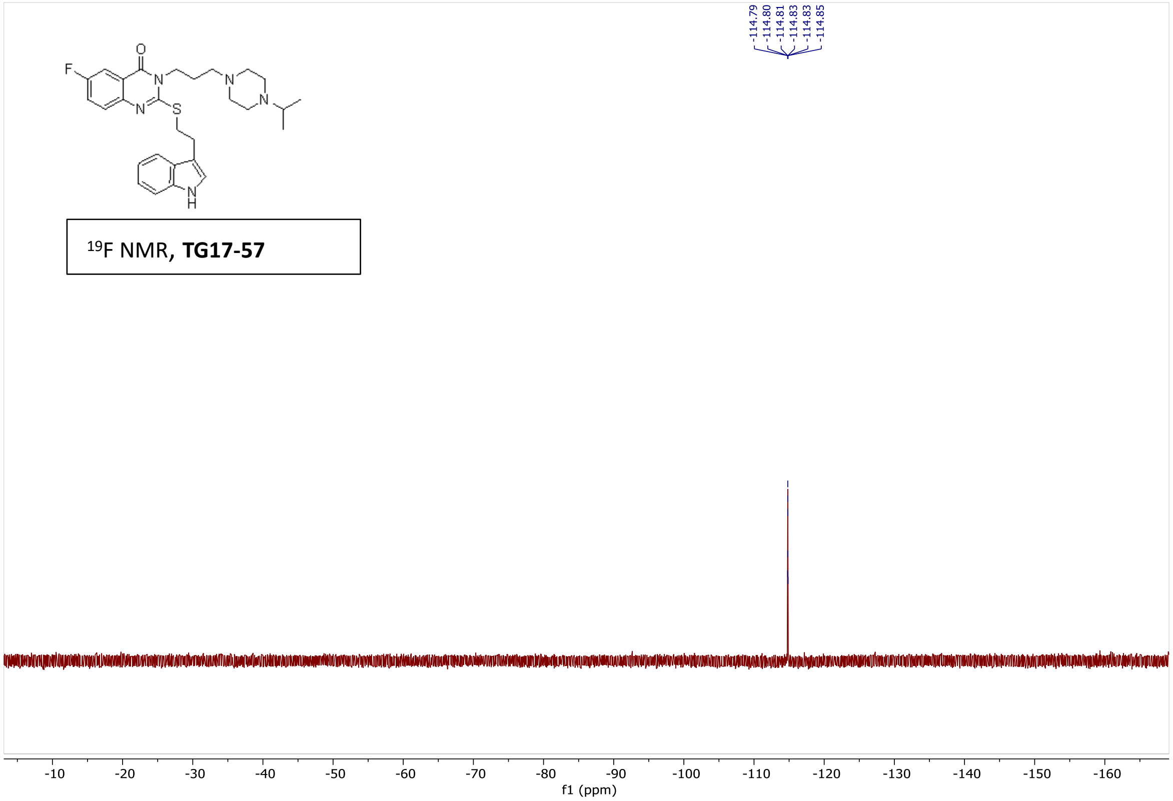
-114.79 -114.80 -114.81 -114.83 -114.83 -114.85
F
O
N
N
S
N
N
N
H
f1 (ppm)

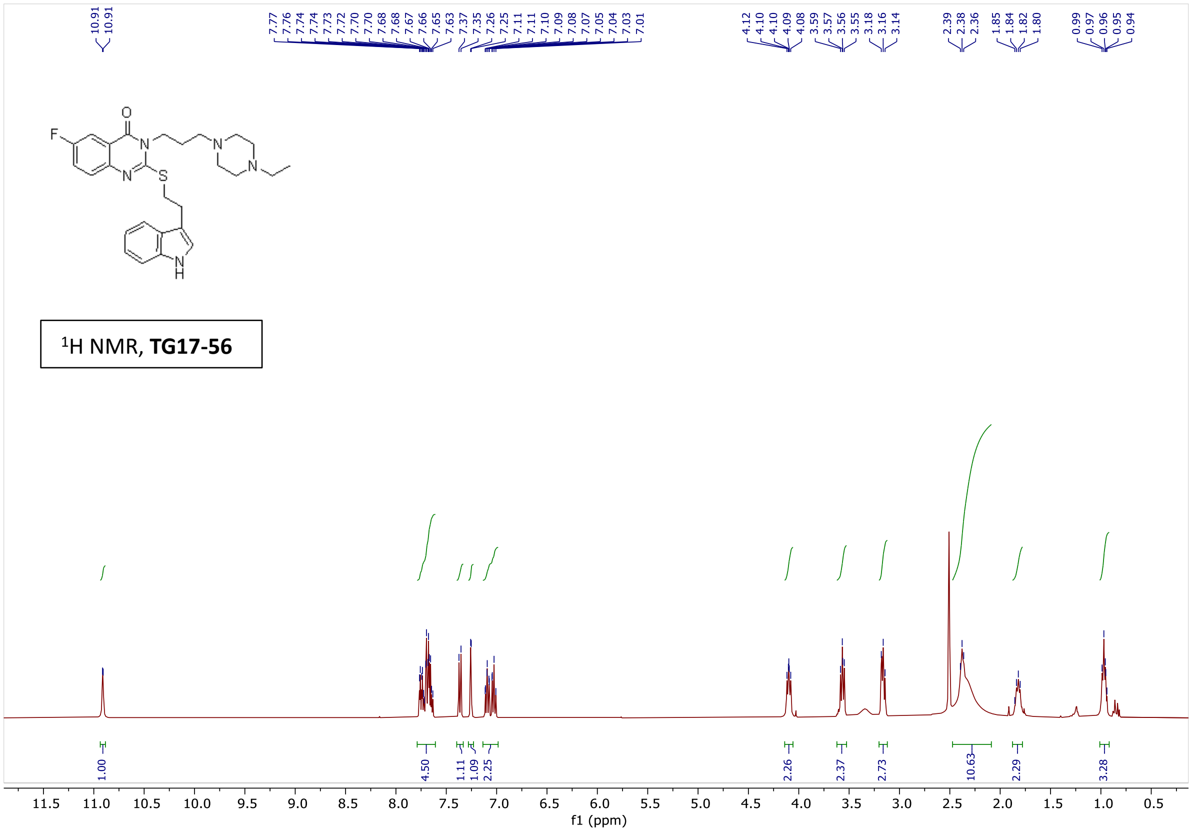

^{1}H NMR, **TG17-56**
f1 (ppm)

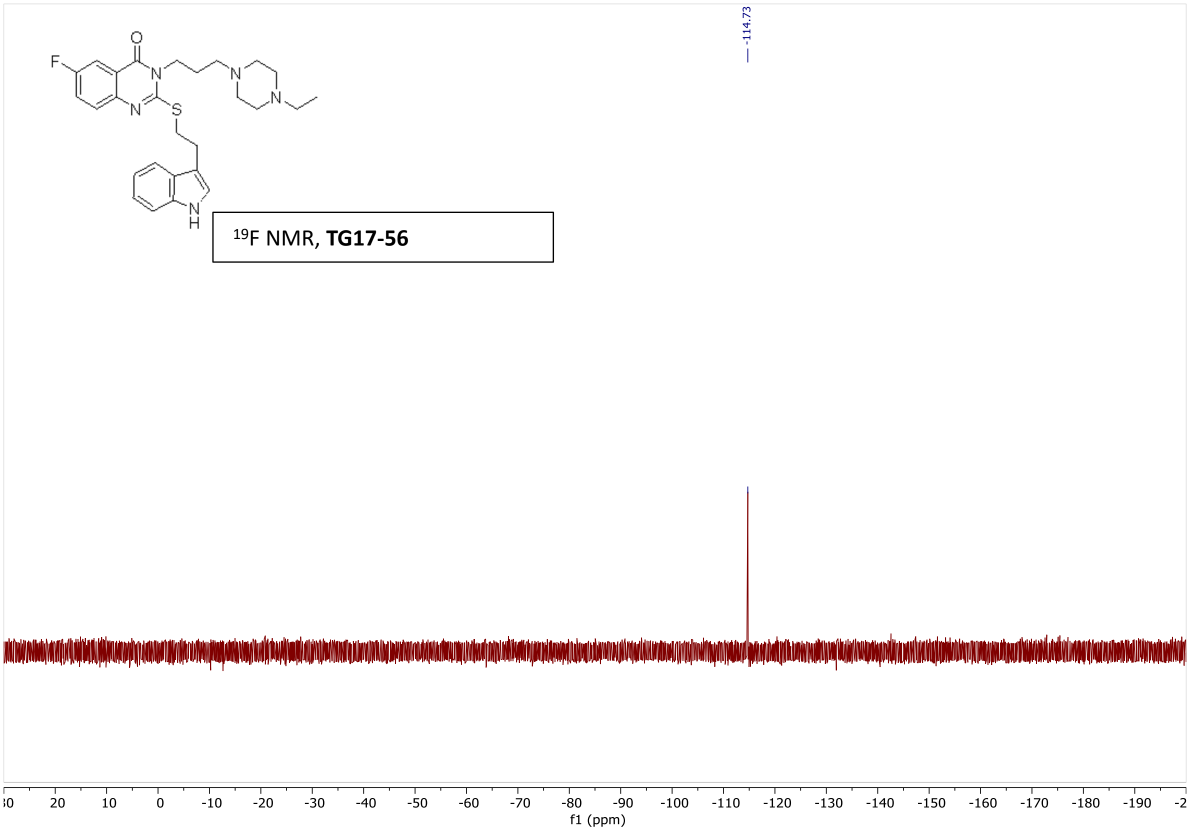

-114.73
^{19}F NMR, **TG17-56**
f1 (ppm)

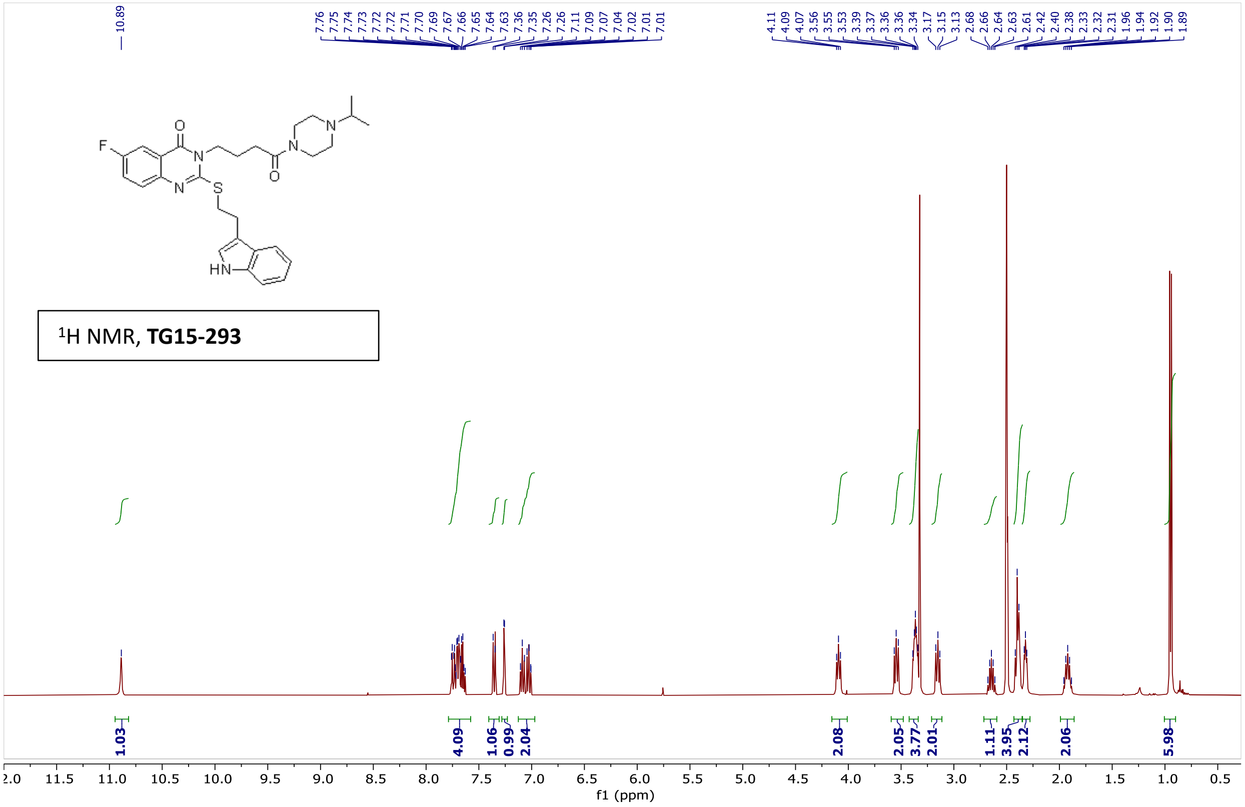
1H NMR, TG15-293
f1 (ppm)

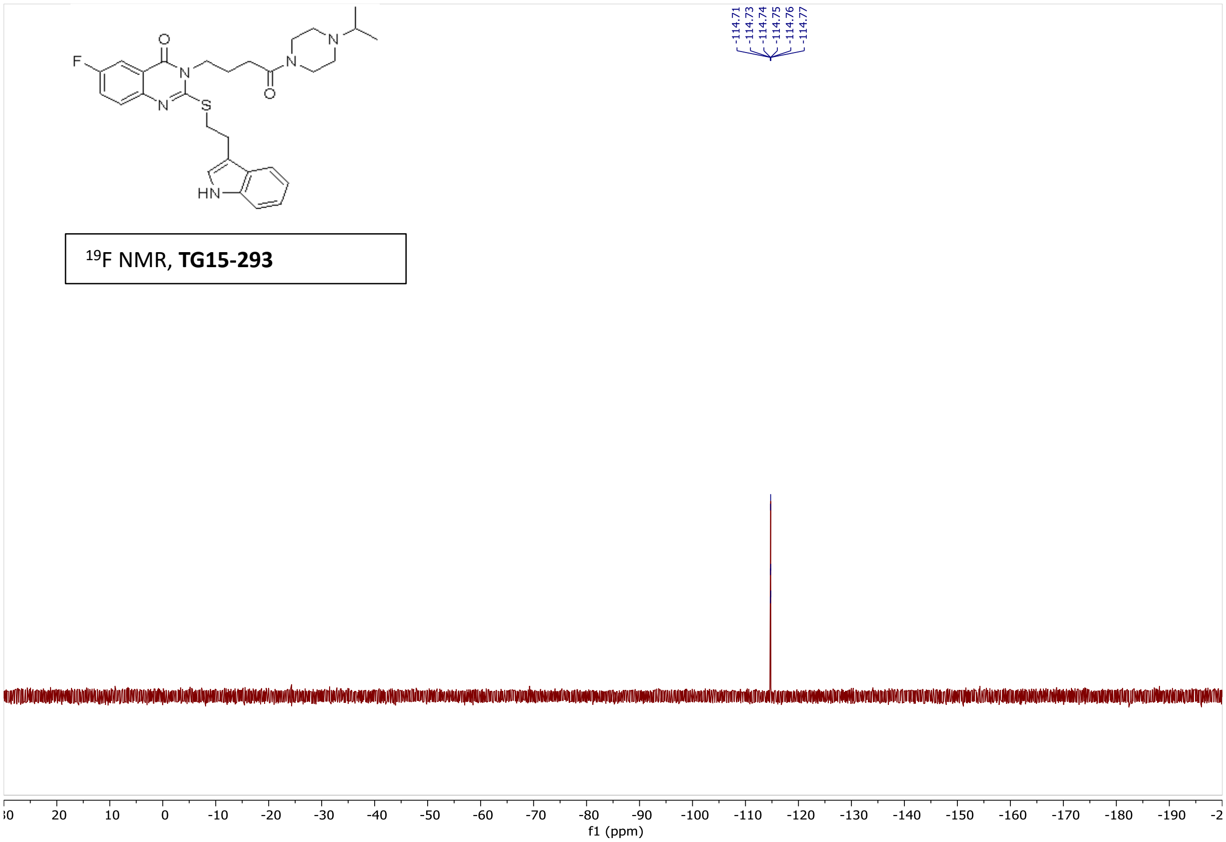
19F NMR, TG15-293
f1 (ppm)

Print of window 80: MS Spectrum
Data File : C:\DATA\RA...2019\OCT-2019\SEQUENCE 2019-10-07 12-27-25\TG15-131-HCl2019-10-07.D
Sample Name : TG15-131-HCl

Acq. Operator : SYSTEM — Seq. Line : 2
Sample Operator : SYSTEM
A[illegible]. Instrument : LCMSD — Location : P1-F8
[illegible]ection Date : 10/7/2019 12:37:01 PM — Inj : 1
Inj Volume : 5.000 µl
Acq. Method : C:\Data\RADHIKA2019\oct-2019\Sequence 2019-10-07 12-27-25\TG-MODERATE-POLAR.M
Last changed : 10/19/2018 4:31:18 PM by SYSTEM
Analysis Method : C:\USERS\PUBLIC\DOCUMENTS\CHEMSTATION\1\METHODS\AgilentGradient.M
Last changed : 7/31/2019 10:45:02 AM by SYSTEM
Method Info : none

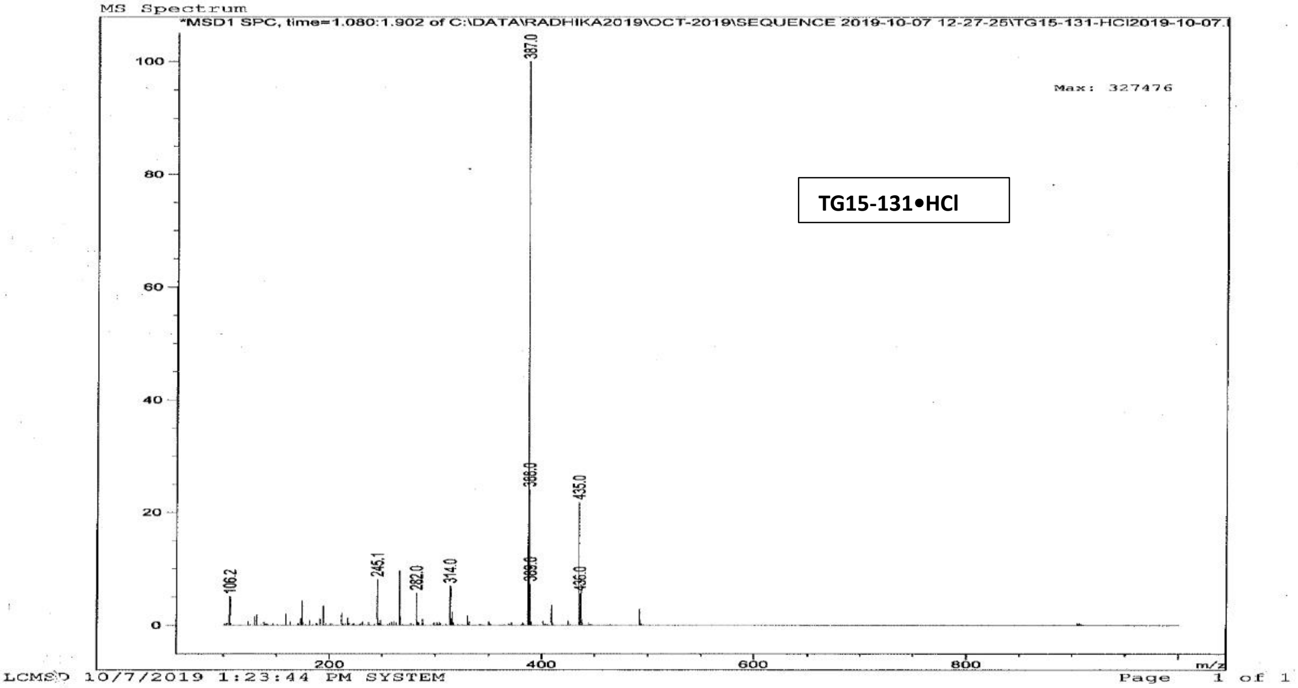

LCMSD 10/7/2019 1:23:44 PM SYSTEM — Page 1 of 1

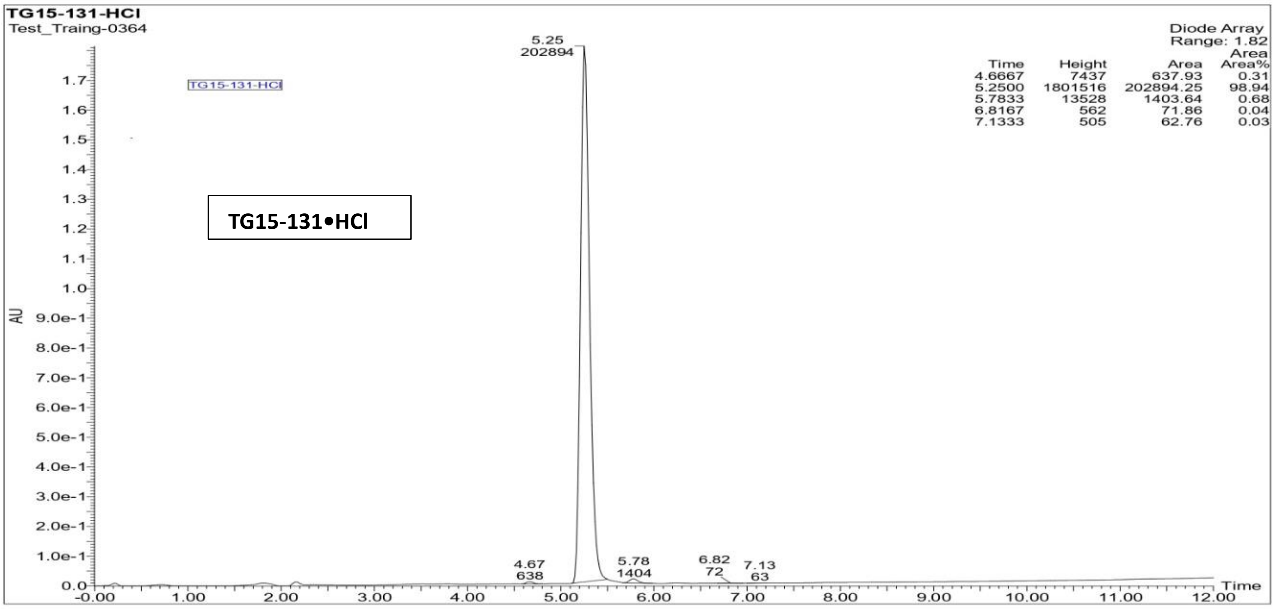

Print of window 80: MS Spectrum
Data File : C:\DATA\RA[illegible]-HCl2019-10-07.D
Sample Name : TG15-124-HCl

Acq. Operator : [illegible]
Sample Operator : [illegible]
Instrument : LCMSD Location : P1-F9
Injection Date : 10/7/2019 12:28:17 PM Inj : 1
Inj Volume : 5.000 µl
Acq. Method : C:\Data\RADHIKA2019\oct-2019\Sequence 2019-10-07 12-27-25\TG-MODERATE-POLAR.M
Last changed : 10/19/2018 4:11:18 PM by SYSTEM
Analysis Method : C:\USERS\PUBLIC\DOCUMENTS\CHEMSTATION\1\METHODS\AgilentGradient.M
Last changed : 7/31/2019 10:45:02 AM by SYSTEM
Method Info : none

MS Spectrum

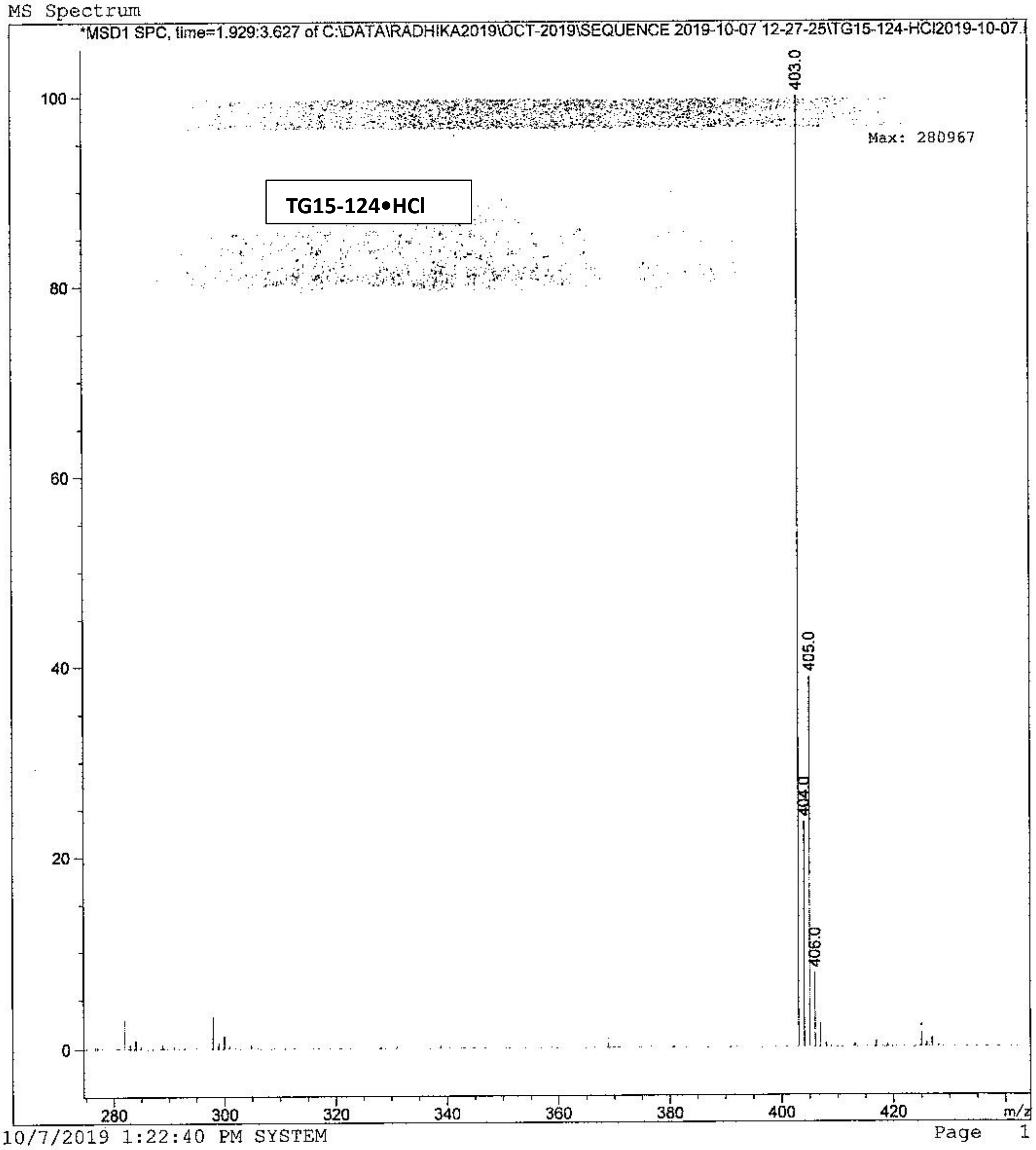

LCMSD 10/7/2019 1:22:40 PM SYSTEM Page 1 of 1

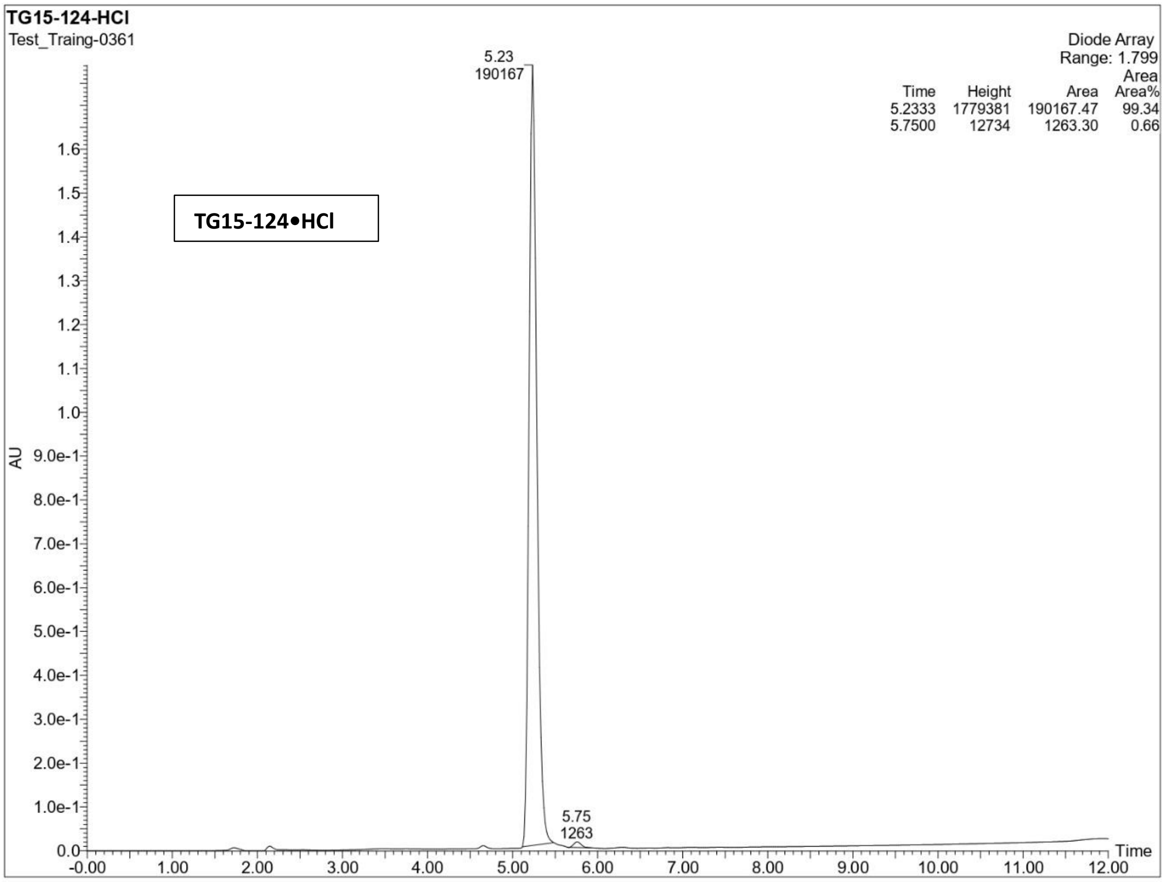
TG15-124-HCl
Test_Traing-0361
Diode Array
Range: 1.799
Time Height Area Area%
5.2333 1779381 190167.47 99.34
5.7500 12734 1263.30 0.66
5.23
190167
5.75
1263
TG15-124•HCl
AU
Time

Print of window 80: MS Spectrum
Data File : C:\DATA\RA...2019\OCT-2019\SEQUENCE 2019-10-07 12-27-25\TG15-132-HCl2019-10-07.D
Sample Name : TG15-132-HCl

Acq. Operator	: SYSTEM	Seq. Line :	3
Sample Operator	: SYSTEM		
Acq. Instrument	: LCMSD	Location :	P1-F7
Injection Date	: 10/7/2019 12:45:34 PM	Inj :	1
		Inj Volume :	5.000 µl

Acq. Method : C:\Data\RADHIKA2019\oct-2019\Sequence 2019-10-07 12-27-25\TG-MODERATE-POLAR.M
Last changed : 10/19/2018 4:31:18 PM by SYSTEM
Analysis Method : C:\USERS\PUBLIC\DOCUMENTS\CHEMSTATION\1\METHODS\AgilentGradient.M
Last changed : 7/31/2019 10:45:02 AM by SYSTEM
Method Info : none

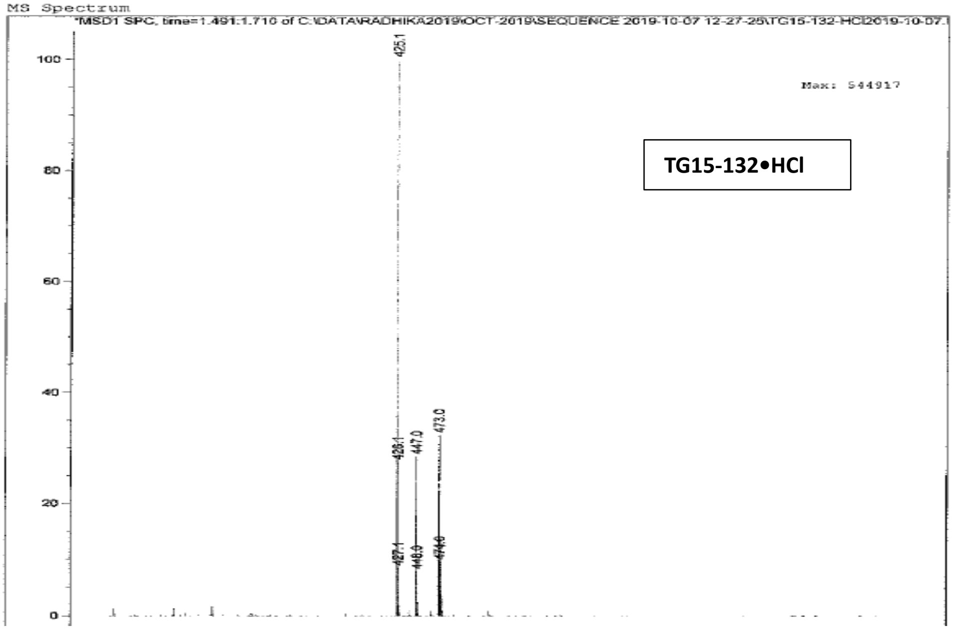

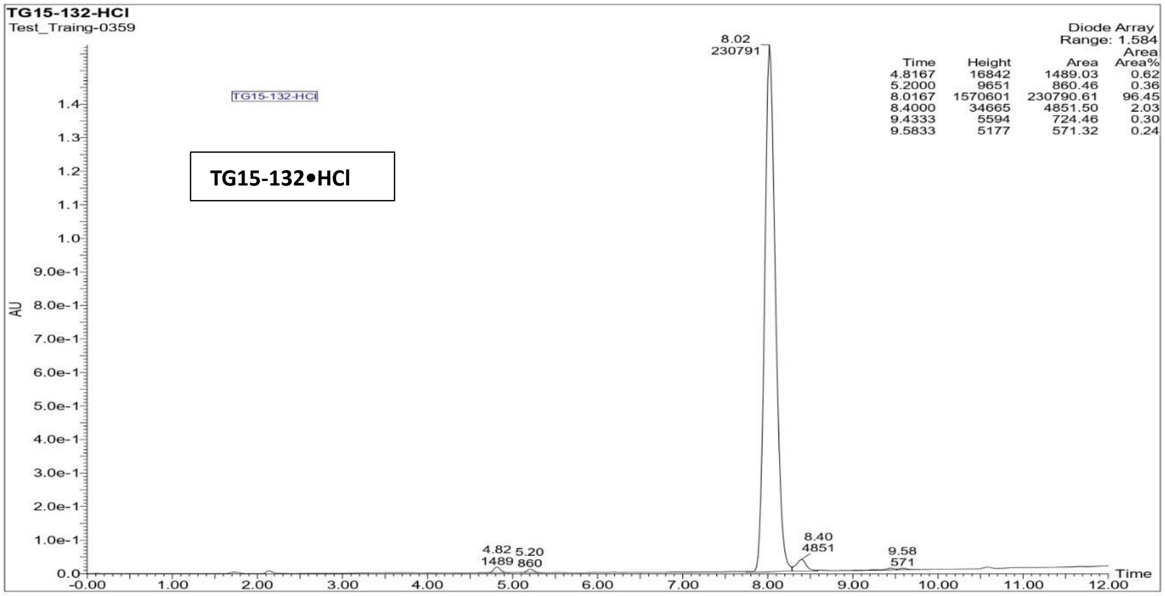

Print of window 80: MS Spectrum
Data File : C:\DATA\RA...2019\OCT-2019\SEQUENCE 2019-10-07 12-27-25\TG15-139-HCl2019-10-07.D
Sample Name : TG15-139-HCl

Acq. Operator : SYSTEM		Seq. Line :	4
Sample Operator : SYSTEM			
A[illegible]. Instrument : LCMSD		Location :	P1-F6
[illegible]ection Date : 10/7/2019 12:54:16 PM		Inj :	1
		Inj Volume :	5.000 µl

Acq. Method : C:\Data\RADHIKA2019\oct-2019\Sequence 2019-10-07 12-27-25\TG-MODERATE-POLAR.M
Last changed : 10/19/2018 4:31:18 PM by SYSTEM
Analysis Method : C:\USERS\PUBLIC\DOCUMENTS\CHEMSTATION\1\METHODS\AgilentGradient.M
Last changed : 7/31/2019 10:45:02 AM by SYSTEM
Method Info : none

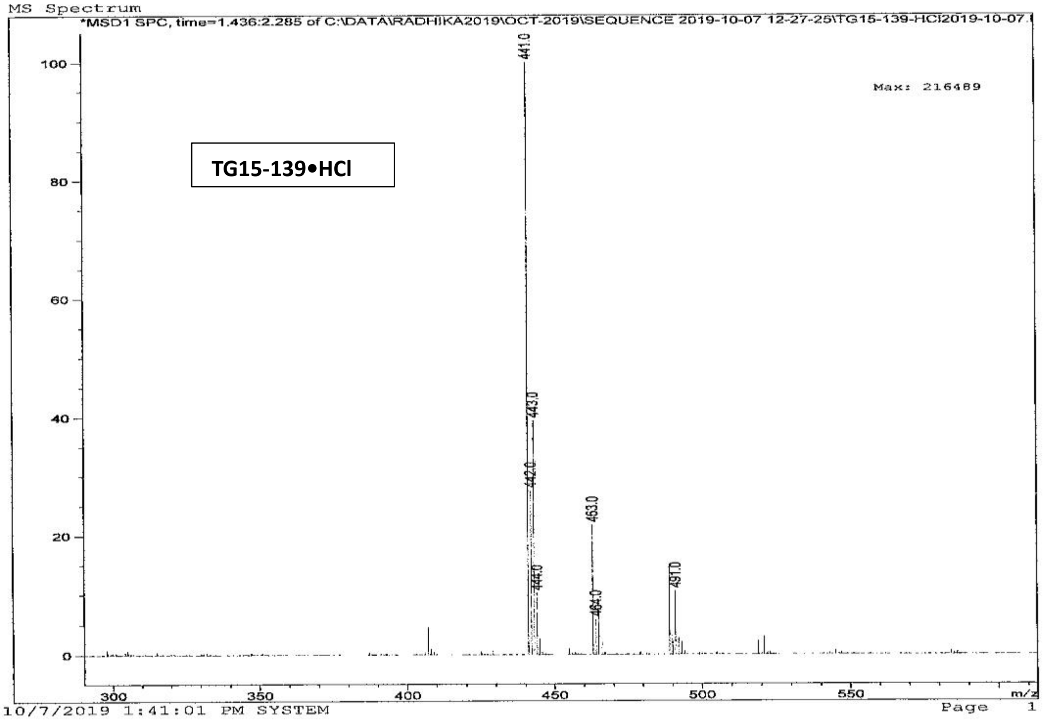

LCMSD 10/7/2019 1:41:01 PM SYSTEM Page 1 of 1

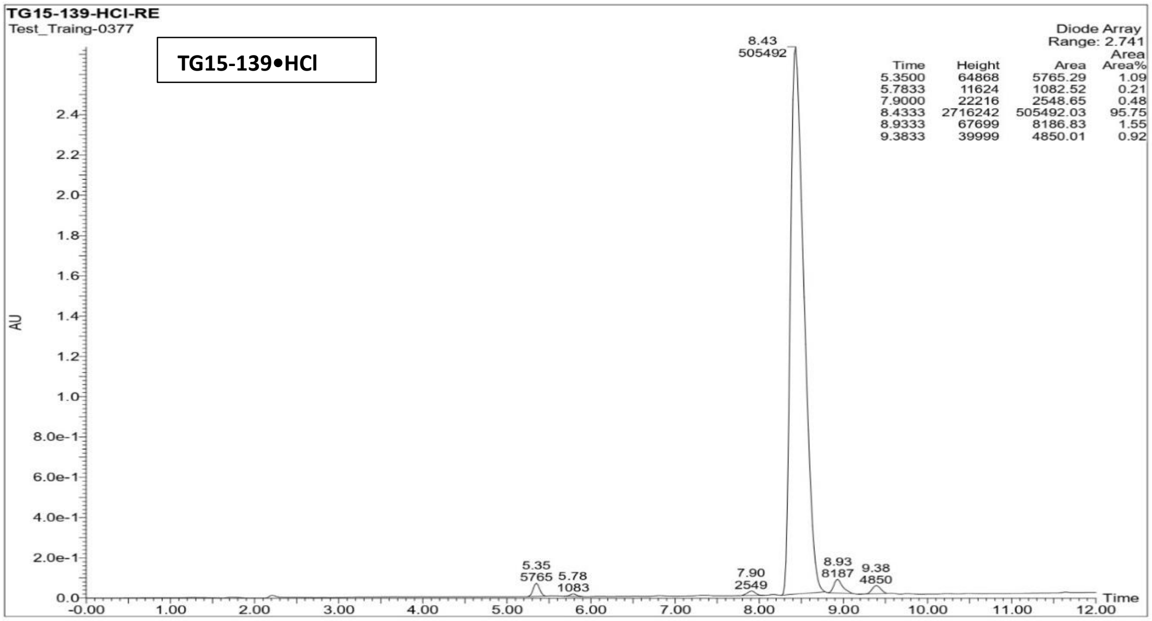
TG15-139-HCl-RE
Test_Traing-0377
TG15-139•HCl
Diode Array
Range: 2.741
Time Height Area Area Area%
5.3500 64868 5765.29 1.09
5.7833 11624 1082.52 0.21
7.9000 22216 2548.65 0.48
8.4333 2716242 505492.03 95.75
8.9333 67699 8186.83 1.55
9.3833 39999 4850.01 0.92
8.43
505492
5.35
5765
5.78
1083
7.90
2549
8.93
8187
9.38
4850
AU
Time

```
Print of window 80: MS Spectrum
Data File  : C:\DATA\RA...AN-2022\JIHO 2022-01-12 12-52-39\TG17-55-2HCl_2022-01-12_13-31-09.D
Sample Name : TG17-55-2HCl
=====================================================================
Acq. Operator   : SYSTEM                         Seq. Line :   4
Sample Operator : SYSTEM
Acq. Instrument : LCMSD                           Location :   P1-F6
Injection Date  : 1/12/2022 1:31:51 PM                 Inj :   1
                                                Inj Volume : 5.000 µl
Acq. Method     : C:\Data\RADHIKA2021\jan-2022\Jiho 2022-01-12 12-52-39\Chang_002_ACN_20_100_
                  12min_140_750mass.M
Last changed    : 10/8/2021 3:43:44 PM by SYSTEM
Analysis Method : C:\USERS\PUBLIC\DOCUMENTS\CHEMSTATION\1\METHODS\Chang_002_ACN_70_100_8min_
                  140_750mass.M
Last changed    : 1/24/2022 12:53:53 PM by SYSTEM
                  (modified after loading)
```

MS Spectrum

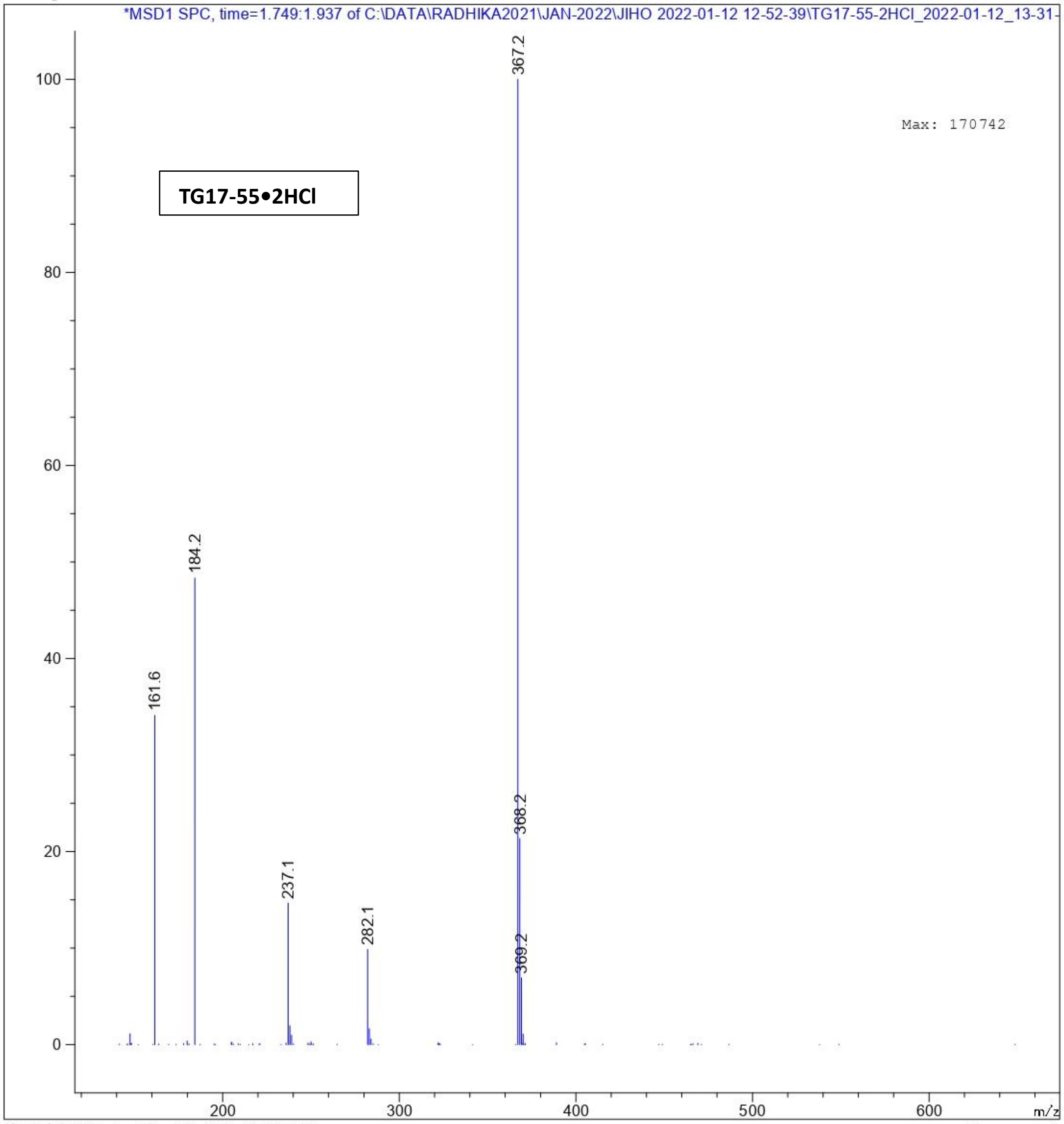

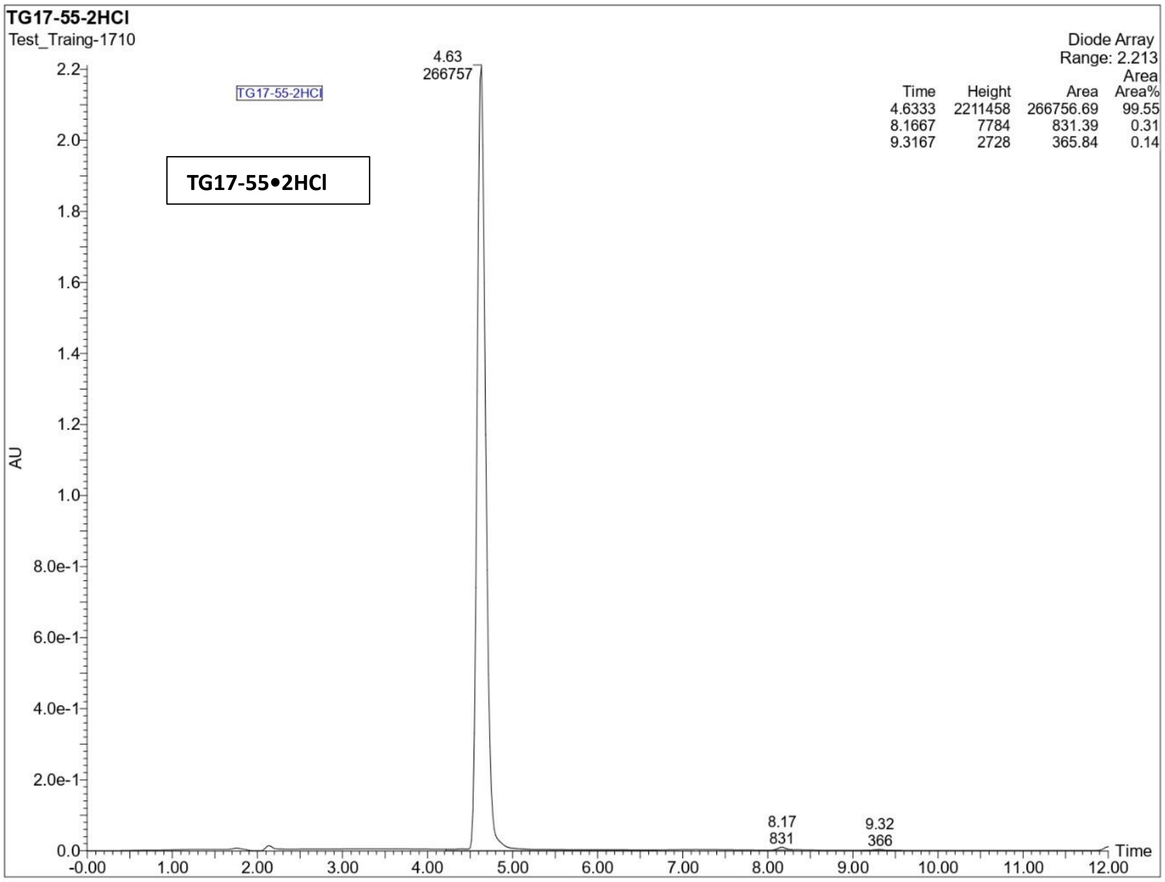

TG17-55-2HCl
Test_Traing-1710
Diode Array
Range: 2.213
Time Height Area Area%
4.6333 2211458 266756.69 99.55
8.1667 7784 831.39 0.31
9.3167 2728 365.84 0.14
4.63
266757
TG17-55-2HCl
TG17-55•2HCl
8.17
831
9.32
366
AU
Time

```
Print of window 80: MS Spectrum
Data File  : C:\DATA\RA...021\JAN-2022\JIHO 2022-01-12 12-52-39\TG17-57_2022-01-12_13-56-41.D
Sample Name : TG17-57
======================================================================
Acq. Operator   : SYSTEM                        Seq. Line :   6
Sample Operator : SYSTEM
Acq. Instrument : LCMSD                          Location :   P1-F4
Injection Date  : 1/12/2022 1:57:21 PM                Inj :   1
                                               Inj Volume : 5.000 µl
Acq. Method     : C:\Data\RADHIKA2021\jan-2022\Jiho 2022-01-12 12-52-39\Chang_002_ACN_20_100_
                  12min_140_750mass.M
Last changed    : 10/8/2021 3:43:44 PM by SYSTEM
Analysis Method : C:\USERS\PUBLIC\DOCUMENTS\CHEMSTATION\1\METHODS\Chang_002_ACN_70_100_8min_
                  140_750mass.M
Last changed    : 1/24/2022 12:53:53 PM by SYSTEM
                  (modified after loading)
```

MS Spectrum

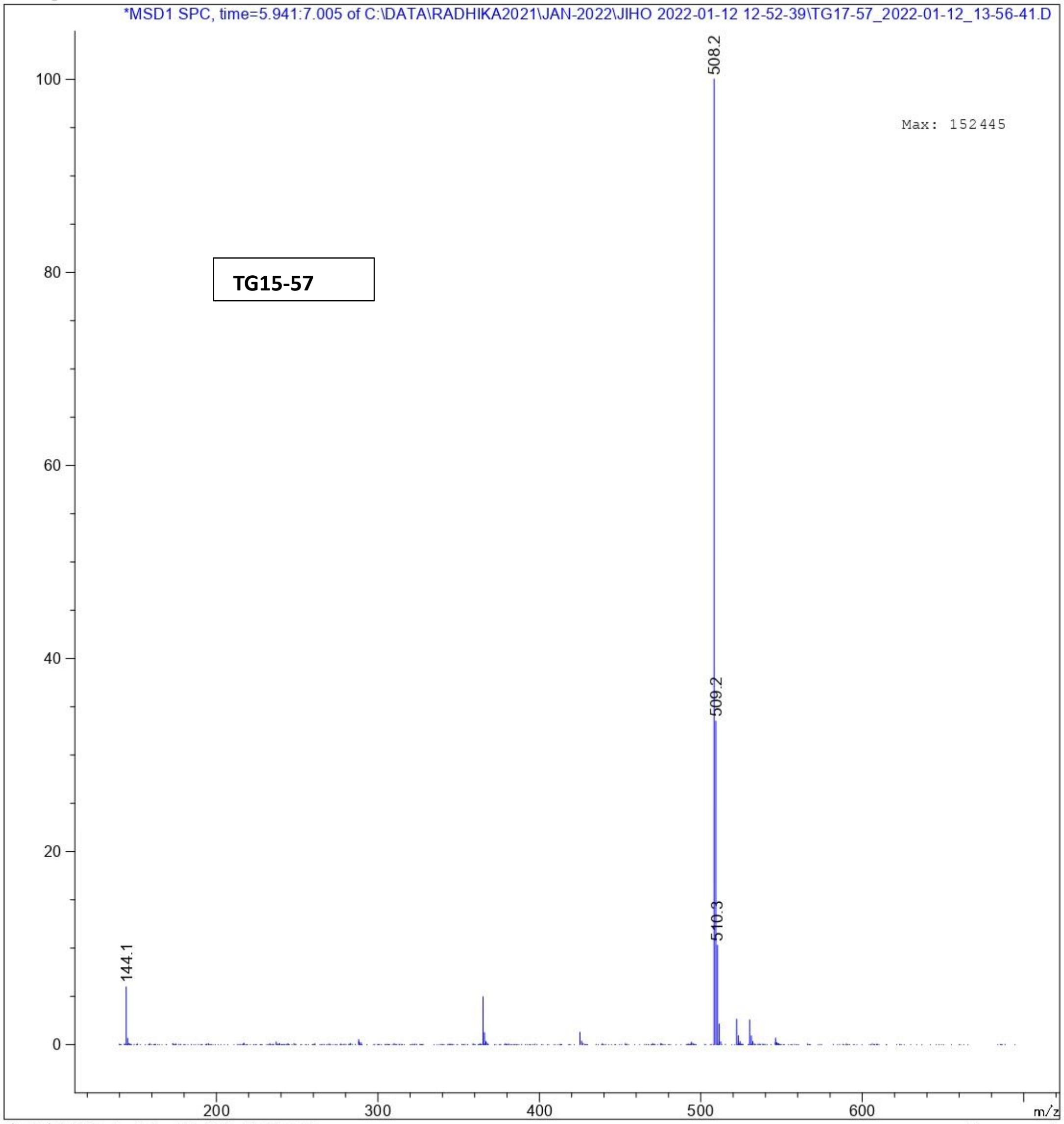

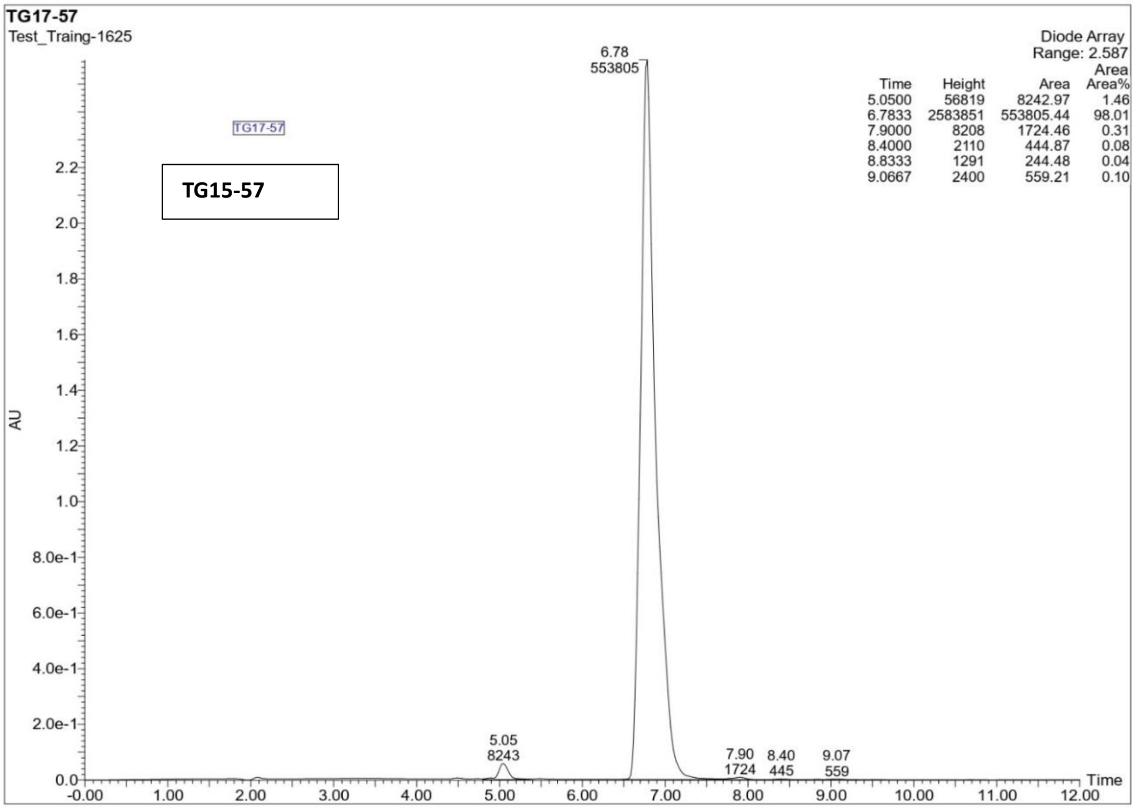
TG17-57
Test_Traing-1625
Diode Array
Range: 2.587
6.78
553805
Time Height Area Area Area%
5.0500 56819 8242.97 1.46
6.7833 2583851 553805.44 98.01
7.9000 8208 1724.46 0.31
8.4000 2110 444.87 0.08
8.8333 1291 244.48 0.04
9.0667 2400 559.21 0.10
TG17-57
TG15-57
AU
5.05
8243
7.90
1724
8.40
445
9.07
559
Time

```
Print of window 80: MS Spectrum
Data File  : C:\DATA\RA...021\JAN-2022\JIHO 2022-01-12 12-52-39\TG17-56_2022-01-12_13-43-55.D
Sample Name : TG17-56
=====================================================================
Acq. Operator   : SYSTEM                         Seq. Line :   5
Sample Operator : SYSTEM
Acq. Instrument : LCMSD                           Location :   P1-F5
Injection Date  : 1/12/2022 1:44:35 PM                 Inj :   1
                                                Inj Volume : 5.000 µl
Acq. Method     : C:\Data\RADHIKA2021\jan-2022\Jiho 2022-01-12 12-52-39\Chang_002_ACN_20_100_
                  12min_140_750mass.M
Last changed    : 10/8/2021 3:43:44 PM by SYSTEM
Analysis Method : C:\USERS\PUBLIC\DOCUMENTS\CHEMSTATION\1\METHODS\Chang_002_ACN_70_100_8min_
                  140_750mass.M
Last changed    : 1/24/2022 12:53:53 PM by SYSTEM
                  (modified after loading)
```

MS Spectrum

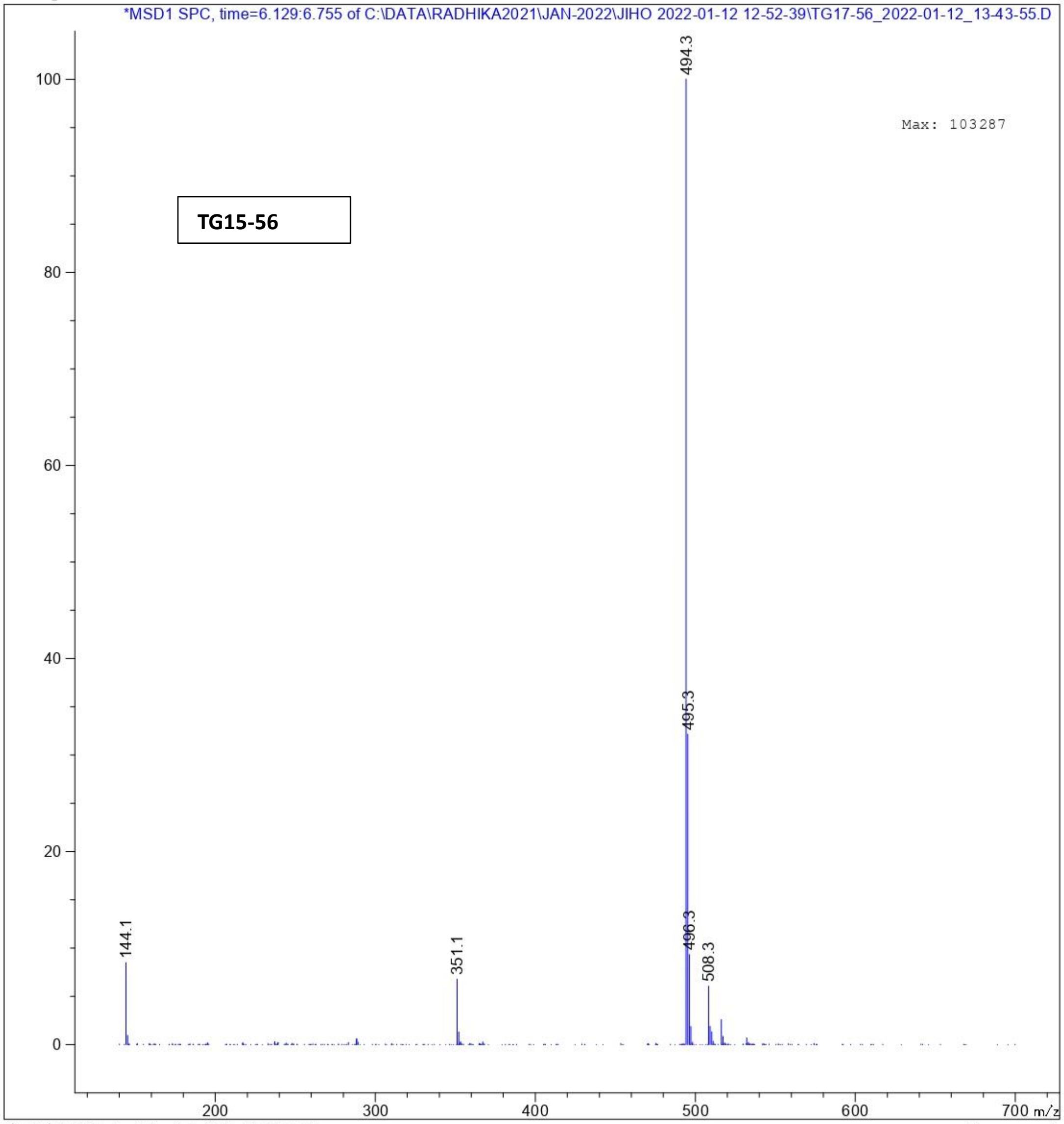

LCMSD 1/24/2022 1:12:24 PM SYSTEM Page 1 of 1

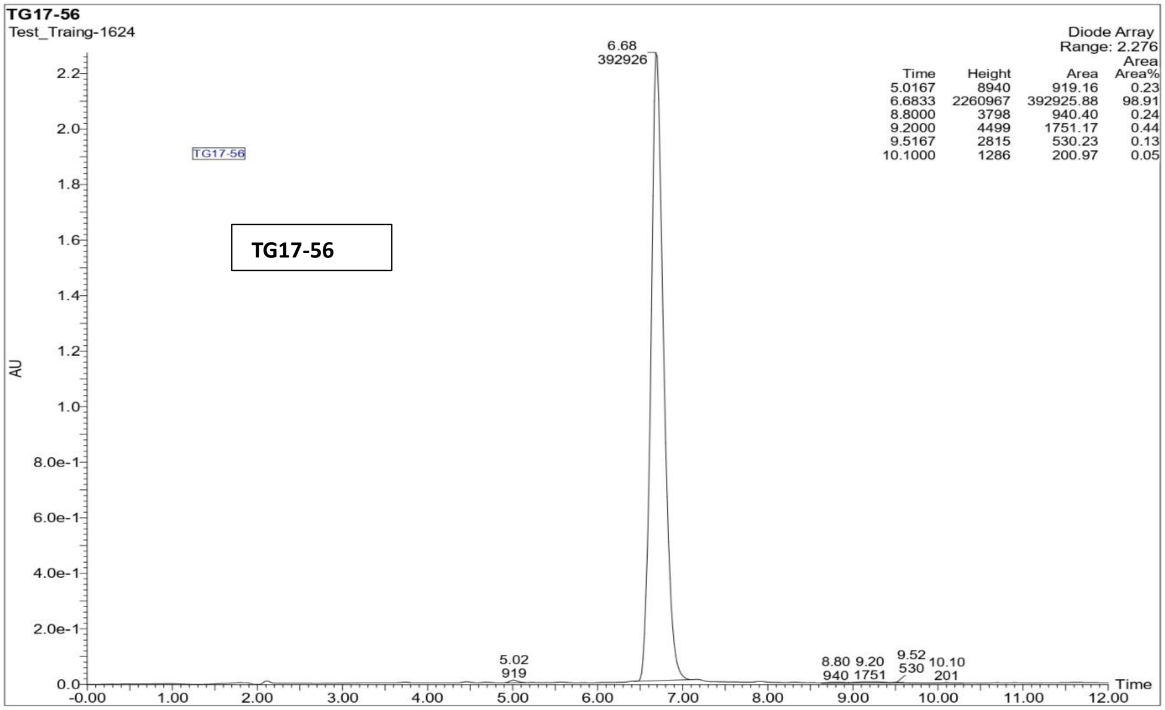
TG17-56
Test_Traing-1624
Diode Array
Range: 2.276
Time Height Area Area%
5.0167 8940 919.16 0.23
6.6833 2260967 392925.88 98.91
8.8000 3798 940.40 0.24
9.2000 4499 1751.17 0.44
9.5167 2815 530.23 0.13
10.1000 1286 200.97 0.05
6.68
392926
TG17-56
TG17-56
AU
5.02
919
8.80 9.20
940 1751
9.52
530
10.10
201
Time

```
Print of window 80: MS Spectrum
Data File  : C:\DATA\RA...21\JAN-2022\JIHO 2022-01-12 12-52-39\TG15-293_2022-01-12_13-18-18.D
Sample Name : TG15-293
=====================================================================
Acq. Operator   : SYSTEM                        Seq. Line :   3
Sample Operator : SYSTEM
Acq. Instrument : LCMSD                          Location :   P1-F7
Injection Date  : 1/12/2022 1:19:03 PM                Inj :   1
                                               Inj Volume : 5.000 µl
Acq. Method     : C:\Data\RADHIKA2021\jan-2022\Jiho 2022-01-12 12-52-39\Chang_002_ACN_20_100_
                  12min_140_750mass.M
Last changed    : 10/8/2021 3:43:44 PM by SYSTEM
Analysis Method : C:\USERS\PUBLIC\DOCUMENTS\CHEMSTATION\1\METHODS\Chang_002_ACN_70_100_8min_
                  140_750mass.M
Last changed    : 1/24/2022 12:53:53 PM by SYSTEM
                  (modified after loading)
```

MS Spectrum

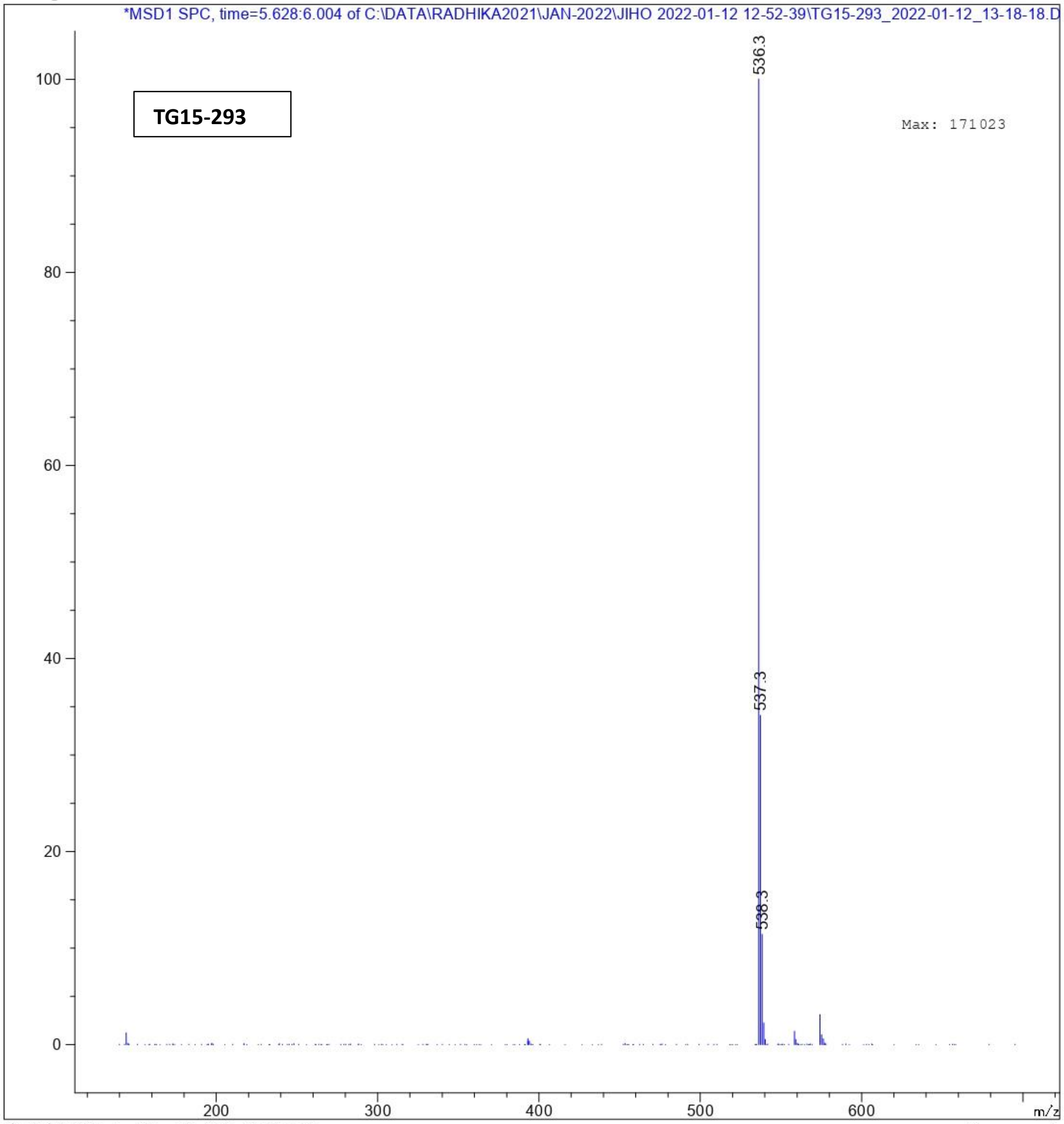

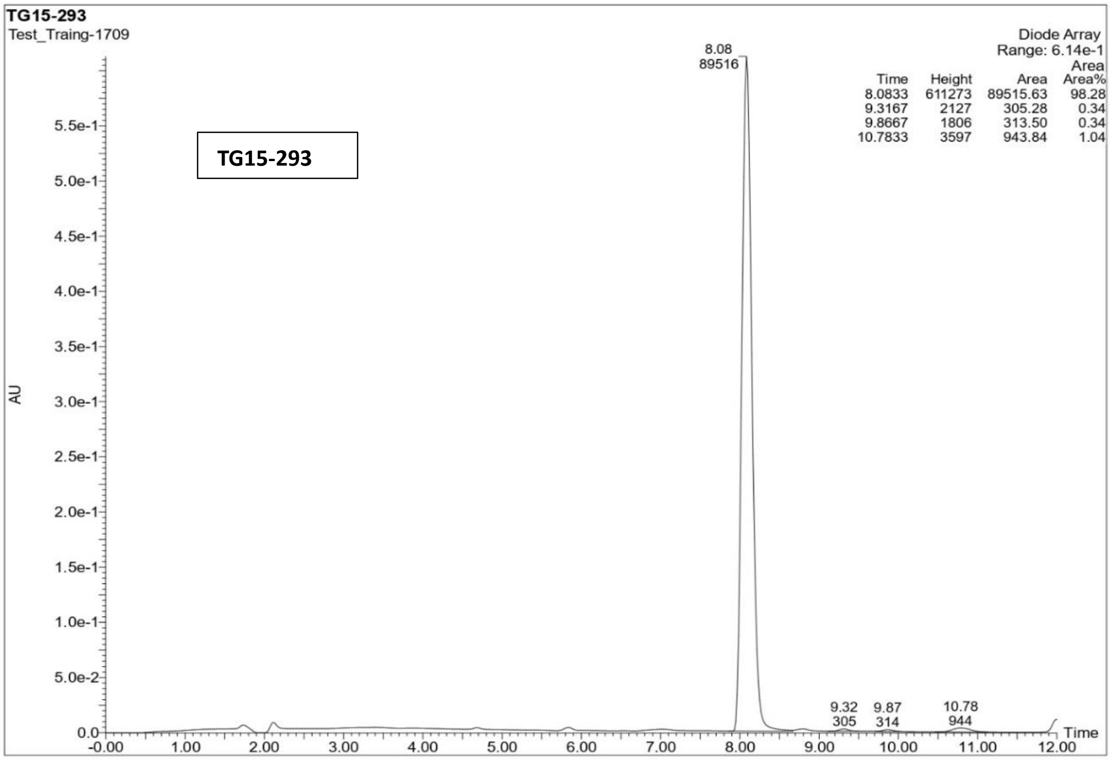
TG15-293
Test_Traing-1709
Diode Array
Range: 6.14e-1
8.08
89516
Time Height Area Area%
8.0833 611273 89515.63 98.28
9.3167 2127 305.28 0.34
9.8667 1806 313.50 0.34
10.7833 3597 943.84 1.04
TG15-293
AU
9.32 305
9.87 314
10.78 944
Time
